# Caloric restriction of db/db mice reverts hepatic steatosis and body weight with divergent hepatic metabolism

**DOI:** 10.1038/srep30111

**Published:** 2016-07-21

**Authors:** Kyung Eun Kim, Youngae Jung, Soonki Min, Miso Nam, Rok Won Heo, Byeong Tak Jeon, Dae Hyun Song, Chin-ok Yi, Eun Ae Jeong, Hwajin Kim, Jeonghyun Kim, Seon-Yong Jeong, Woori Kwak, Do Hyun Ryu, Tamas L. Horvath, Gu Seob Roh, Geum-Sook Hwang

**Affiliations:** 1 Department of Anatomy and Convergence Medical Science, Bio Anti-aging Medical Research Center, Institute of Health Sciences, Gyeongsang National University School of Medicine, Jinju, Republic of Korea; 2 Integrated Metabolomics Research Group, Western Seoul Center, Korea Basic Science Institute, Seoul, Republic of Korea; 3 Department of Chemistry, Sungkyunkwan University, Suwon, Republic of Korea; 4 Department of Biochemistry, University of Nebraska-Lincoln, Lincoln, NE 68588, USA; 5 Department of Pathology, Institute of Health Sciences, Gyeongsang National University School of Medicine, Jinju, Republic of Korea; 6 Department of Medical Genetics, Ajou University School of Medicine, Suwon, Republic of Korea; 7 C&K Genomics, Seoul, Republic of Korea; 8 Program in Integrative Cell Signaling and Neurobiology of Metabolism, Section of Comparative Medicine, Yale University School of Medicine, New Haven, CT 06520, USA; 9 Department of Chemistry and Nano Science, Ewha Womans University, Seoul, Republic of Korea

## Abstract

Non-alcoholic fatty liver disease (NAFLD) is one of the most frequent causes of liver disease and its prevalence is a serious and growing clinical problem. Caloric restriction (CR) is commonly recommended for improvement of obesity-related diseases such as NAFLD. However, the effects of CR on hepatic metabolism remain unknown. We investigated the effects of CR on metabolic dysfunction in the liver of obese diabetic db/db mice. We found that CR of db/db mice reverted insulin resistance, hepatic steatosis, body weight and adiposity to those of db/m mice. ^1^H-NMR- and UPLC-QTOF-MS-based metabolite profiling data showed significant metabolic alterations related to lipogenesis, ketogenesis, and inflammation in db/db mice. Moreover, western blot analysis showed that lipogenesis pathway enzymes in the liver of db/db mice were reduced by CR. In addition, CR reversed ketogenesis pathway enzymes and the enhanced autophagy, mitochondrial biogenesis, collagen deposition and endoplasmic reticulum stress in db/db mice. In particular, hepatic inflammation-related proteins including lipocalin-2 in db/db mice were attenuated by CR. Hepatic metabolomic studies yielded multiple pathological mechanisms of NAFLD. Also, these findings showed that CR has a therapeutic effect by attenuating the deleterious effects of obesity and diabetes-induced multiple complications.

Over the past decade, the prevalence of diabetes has dramatically increased across all genders and age groups and has reached epidemic proportions in developed and developing countries due to increased obesity rates^1^. In particular, non-alcoholic fatty liver disease (NAFLD) and non-alcoholic steatohepatitis (NASH) are features of metabolic syndrome and are strongly associated with insulin resistance, dyslipidemia, obesity, and hyperglycemia leading to type 2 diabetes (T2D)^2^,^3^. In NAFLD, glycerolipids accumulate in the liver (causing hepatic steatosis) due to an imbalance between lipid storage and lipid removal^2^. Also, NAFLD disturbs hepatic lipid and glucose metabolism and causes inflammation in the liver^4^. NASH, a severe form of NAFLD that is accompanied by inflammation and fibrosis, progresses to cirrhosis and hepatic failure^5^. Thus, various pathological changes in genes and proteins, including those that produce metabolites, contribute to the progression of NAFLD.

Caloric restriction (CR) reduces mortality in diverse species from age and other causes, including diabetes, cancer, cardiovascular disease, and brain atrophy^6^,^7^. The effects of CR on lifespan and health span have been known for nearly a century. Generally, CR causes major metabolic reprogramming toward efficient fuel utilization and a reduction in oxidative damage to macromolecules^8^. Although a range of putative mechanisms have been proposed, the precise molecular mechanisms underlying these effects remain unknown^9^.

Previous studies have shown that NAFLD changes the levels of metabolites, proteins and genes in the liver of human^5^,^10^,^11^ and animal models^3^,^6^. In particular, NAFLD causes the accumulation of lipids in the liver and results in inflammation and mitochondrial dysfunction^12^,^13^. It has also been reported that CR alters metabolism; however, these findings have been limited to normal mouse models and confirmed its effect against aging and/or dietary excess^14^,^15^. Moreover, the process of improvement from NAFLD caused by CR treatment is unclear. In this study, we examined alterations in hepatic metabolism caused by CR treatment in the context of NAFLD of db/db mice, to investigate several metabolic pathways related to CR and NAFLD. We also investigated the hypothesis that long-term CR administration protects against NAFLD by inhibiting hepatic steatosis, autophagy, endoplasmic reticulum (ER) stress, mitochondrial fission, inflammation, and collagen deposition.

## Results

### Effects of CR on metabolic parameters and hepatic steatosis in db/db mice

**Figure 1:**
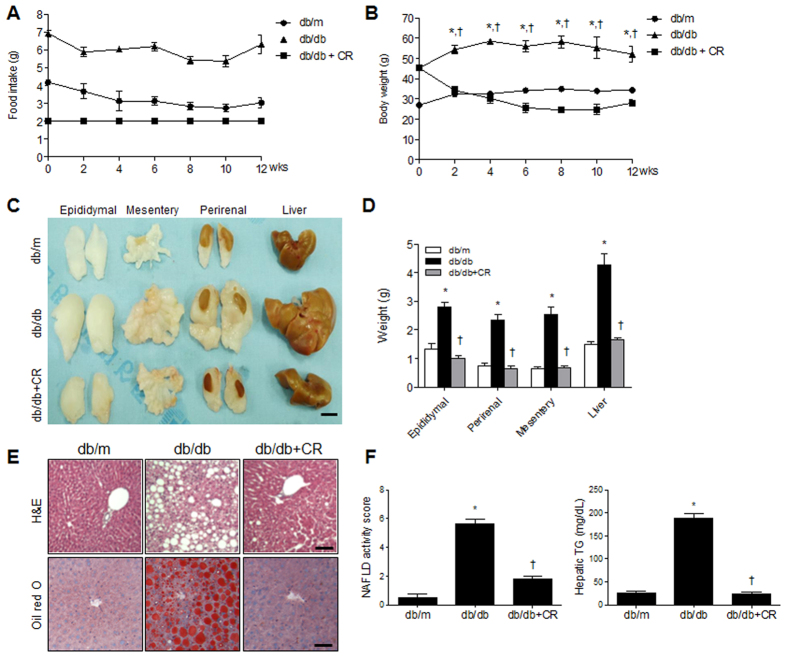
Effects of caloric restriction (CR) on obesity and hepatic steatosis in db/db mice. Food intake (**A**) and body weight (**B**) of db/db and db/m mice. Gross morphology (**C**) and weight (**D**) of intraabdominal fat deposits (epididymal fat pads, mesentery fat, and perirenal fat) and the liver. (**E**) Histological analysis of hepatic fat accumulation by H&E and Oil red O staining. Scale bar, 100 μm. (**F**) NAFLD activity score and concentration of hepatic triglycerides (TGs). Data are shown as the mean ± SEM. *p < 0.05 for db/db versus db/m mice. ^†^*p* < 0.05 for db/db+CR versus db/db mice.

To investigate the effect of CR on obesity and diabetes-induced metabolic disturbances in db/db mice, mice were maintained on the normal standard diet chow (ND) or CR (2 g/day) for 12 weeks (Fig. 1A). The total caloric intake of db/db mice was 85.09 ± 0.86% higher than db/m mice and 123.53 ± 16.47% higher than db/db+CR mice (P < 0.0001) (Fig. S1). Two weeks after CR, the body weight of db/db+CR mice was reduced compared with db/db mice (Fig. 1B). The size and weight of intraabdominal fat deposits and livers of db/db mice decreased after CR (Fig. 1C,D). H&E and Oil Red O staining showed that hepatic steatosis in db/db mice was reduced by CR administration (Fig. 1E). The analysis of histological scoring for NAFLD activity revealed that the liver histology in db/db mice was significantly improved by CR (Fig. 1F). Consistent with the Oil Red O staining, we found that the hepatic triglyceride (TG) concentration, which is higher in db/db mice, was significantly decreased by CR (Fig. 1F). To determine the effects of CR on serum metabolic parameters in db/db mice with or without CR, we measured the concentration of various proteins. As shown in Table 1, hyperinsulinemia, hyperleptinemia, and hypoadiponectinemia in db/db mice were reversed by CR. We also found that hepatic enzymes and total cholesterol were higher in db/db mice than in db/m mice, and were significantly decreased by CR. However, serum glucose, TG, and free fatty acids (FFA) levels in db/db mice were not significantly reduced by CR (Table 1). In particular, the fasting blood glucose levels in db/db mice were not significantly corrected by CR (Fig. S2A). To examine the effect of CR on insulin resistance in db/db mice, we performed an insulin tolerance test (Fig. S2B). Consistent with the effects of CR on serum insulin, the uncontrolled glucose level in db/db mice was reduced by CR (Fig. S2C).

### Metabolomic profiling of the mouse liver using ^1^H NMR

**Figure 2:**
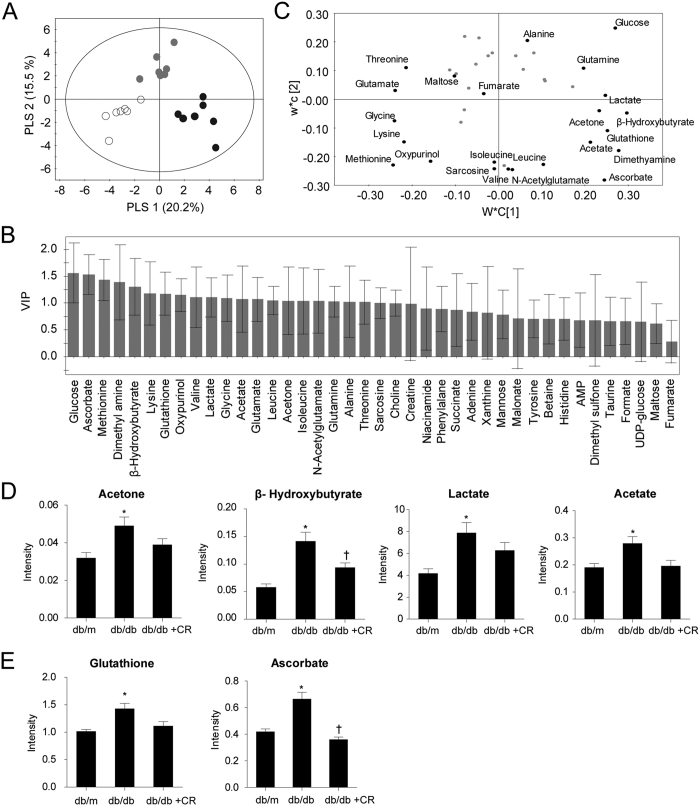
Quantification of aqueous metabolites in liver samples. Score scatter plot (**A**), variable importance of projection (VIP) score plot (**B**), and loading plot (**C**) of the partial least-squares discriminant analysis (PLS-DA) model. Score plot shows a good separation among db/m (circle), db/db (black dots), and db/db+caloric restriction (CR; gray dots) mice. Identified metabolites that have high VIP values (>1) are presented in the loading plot. Quantified metabolites (**D,E**) with high VIP values are related to inflammation, energy, and lipid metabolism. Data are shown as the mean ± SEM. *p < 0.017 for db/db versus db/m mice. ^†^*p* < 0.017 for db/db+CR versus db/db mice.

To investigate aqueous metabolite changes in the liver of db/db mice with or without CR, we performed metabolic profiling using ^1^H nuclear magnetic resonance (^1^H NMR). Representative one-dimensional ^1^H NMR spectra of aqueous liver samples from three different groups are shown in Fig. S3. We quantified 40 metabolites (Table S2) and employed multivariate statistical analysis to compare mouse groups using the partial least squares discriminant analysis (PLS-DA) model derived from the quantification. The PLS-DA score plot (Fig. 2A) showed a remarkable separation of the groups with high goodness of fit and predictability, as indicated by the R^2^ and Q^2^ values, respectively (R^2^Y = 0.986, Q^2^ = 0.993). Permutation tests (Fig. S4) were performed to validate PLS-DA models and the results strongly confirmed the validity of the models. Acetate, acetone, ascorbate, β-hydroxybutyrate, dimethylamine, glutathione, and lactate separated db/db mice from other groups in loading plots and had high variable importance of projection (VIP) values (VIP > 1) (Fig. 2B,C). Among these metabolites, acetate, acetone, β-hydroxybutyrate, and lactate are related to energy and lipid metabolism^16^,^17^, and glutathione and ascorbate are known antioxidants in hepatic inflammation^18^. Quantifications and *p* values of these metabolites are shown in Fig. 2D,E. Ascorbate and β-hydroxybutyrate were significantly (*p* < 0.017) increased in db/db mice compared to db/m mice and decreased in db/db+CR mice. Acetone, acetate, lactate, and glutathione were significantly higher in the db/db mice compared with db/m mice, but the reduction caused by CR was not significant.

### Lipidomic profiling of mouse livers using UPLC-QTOF-MS

**Figure 3:**
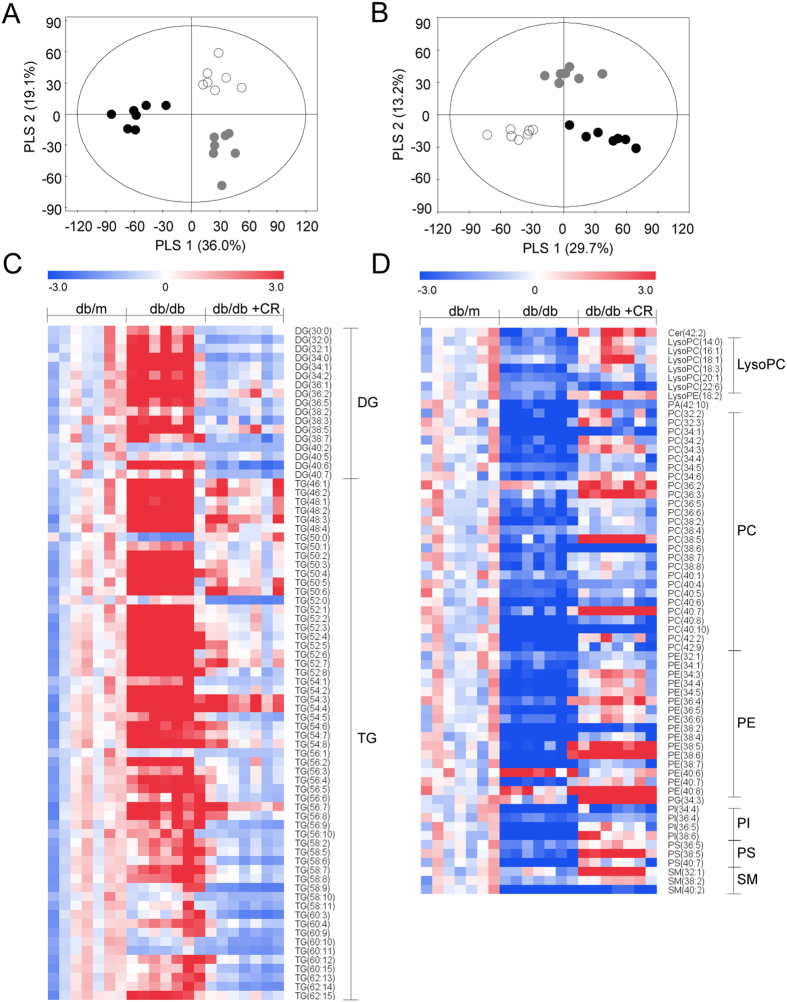
Alteration in lipid species due to caloric restriction (CR) in db/db mice. Partial least-squares discriminant analysis score plots based on the UPLC-QTOF-MS-positive (**A**) and -negative (**B**) ionization mode, from liver samples from db/m (circle), db/db (black dots), and db/db+CR (gray dots) mice. Heatmap shows significant (*p* < 0.017, variable importance of projection >1) glycerolipid (**C**) and phospholipid and sphingolipid (**D**) species altered in db/db+CR versus db/db mice. Each value in the heatmap is a colored representation of a calculated Z-score.

To find lipid species related to inflammation, energy, and lipid metabolism, we applied lipidomic profiling using ultra performance liquid chromatography-quadrupole time of flight-mass spectrometry (UPLC-QTOF-MS). Representative UPLC-QTOF-MS spectra of positive and negative ionization modes obtained from db/m liver tissue are shown in Fig. S5. We applied multivariate statistical analysis using PLS-DA models derived from positive and negative ionization modes, respectively. Each PLS-DA score plot (Fig. 3A,B) showed a clear differentiation in three groups (R^2^Y = 0.986, Q^2^Y = 0.993 for positive ionization mode and R^2^Y = 0.992, Q^2^Y = 0.949 for negative ionization mode), and permutation tests validated the reliability of the model (Fig. S6). We identified 138 significant (*p* < 0.017, VIP > 1) lipid species (119 in positive and 19 in negative ionization modes) that have different intensities in db/db versus db/db+CR as shown in heatmaps. Most glycerolipids [diacylglycerol (DG) and TG] were higher in db/db mice than db/m mice and were decreased by CR. Some glycerolipids, such as TG and DG with longer acyl chains (carbon numbers ≥58 and ≥40, respectively), were not different between db/m and db/db mice (Fig. 3C). Glycerolphospholipids [lysophosphatidylcholine (lysoPC), lysophosphatidylethanolamine (lysoPE), phosphatidic acid (PA), phosphatidylcholine (PC), phosphatidylethanolamine (PE), phosphatidylglycerol (PG), phosphatidylinositol (PI), and phosphatidylserine (PS)] and sphingolipids [ceramide (Cer) and sphingomyelin (SM)] mostly decreased in db/db mice compared with the db/m mice, except PE (40:6). Glycerolphospholipid and sphingolipid levels in the db/db+CR model were mostly increased compared to the db/db model (Fig. 3D).

### Effects of CR on TG synthesis and lipogenesis in the livers of db/db mice

**Figure 4:**
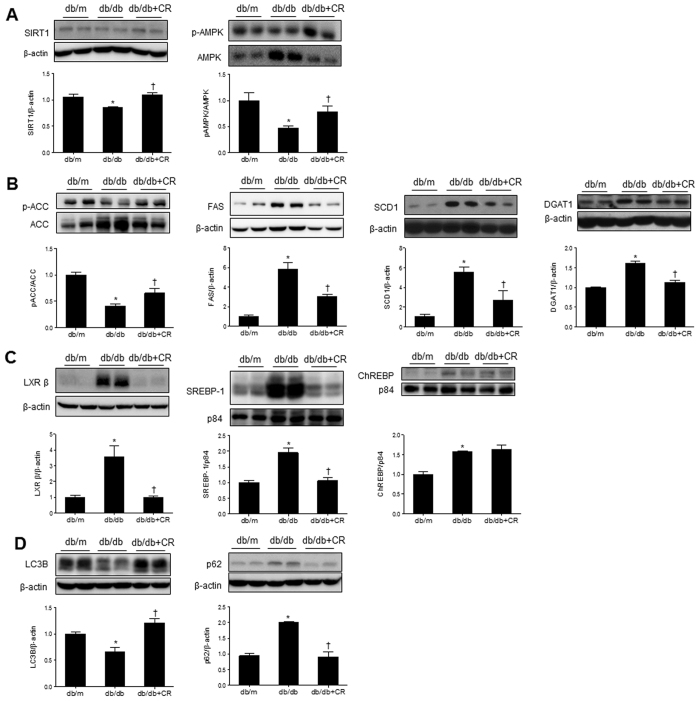
Effects of caloric restriction (CR) on triglyceride synthesis-related lipogenesis and autophagy in the livers of db/db mice. Western blots and quantifications showing expression levels of hepatic SIRT1 and p-AMPK/AMPK (**A**), p-ACC/ACC, FAS, SCD1, and DGAT1 (**B**), LXR β, SREBP-1, and ChREBP (**C**), and autophagy-related LC3B and P62 (**D**). Band intensity was normalized to β-actin, p84, or VDAC1. Data are shown as the mean ± SEM. *p < 0.05 for db/db versus db/m mice. ^†^*p* < 0.05 for db/db+CR versus db/db mice.

Lipid accumulation can increase as a result of increased fat synthesis, reduced fat oxidation, increased delivery of FFA from peripheral adipose tissues to the liver, or enhanced de novo lipogenesis in the liver itself^19^. Sirtuin 1 (SIRT1), which is activated in fasted state, phosphorylates AMPK and enhances energy metabolism. We found that decreased hepatic SIRT1 expression in db/db mice was reversed by CR, which increased AMPK activity (Fig. 4A).

The synthesis of TG in the liver is nutritionally regulated by key metabolic enzymes^19^. As shown in Fig. 1F and Table S2, we found excessive accumulation of serum and hepatic TG in db/db mice. Western blotting showed that lipogenic proteins [acetyl-CoA carboxylase (ACC), fatty acid synthase (FAS), stearoyl-coenzyme A desaturase 1 (SCD1), and diacylglycerol acyltransferase 1 (DGAT1)], which are the key regulators of lipid synthesis in db/db mice, were increased compared to db/m mice. However, these were significantly reversed by CR (Fig. 4B). In particular, these lipogenic enzymes are transcriptionally regulated by sterol regulatory element-binding protein 1 (SREBP-1), carbohydrate response element binding protein (ChREBP), and liver X receptor (LXR) in the liver. We found that CR inhibits hepatic LXRβ and SREBP-1 expression in db/db mice, while increased ChREBP in the liver of db/db mice was not reversed by CR (Fig. 4C). In particular, it is important to remove the excessive accumulation of hepatic TG through autophagy. We performed western blots for light chain 3 beta (LC3B) and p62 in the livers of db/db mice (Fig. 4D). The hepatic LC3B level was significantly reduced in db/db mice, and a significant increase in db/db+CR mice was observed. In contrast, the increased p62 expression level in db/db mice was significantly decreased by CR. These data indicate that hepatic steatosis-induced defective autophagy is improved by CR.

### Effects of CR on ketogenesis and mitochondrial biogenesis in the livers of db/db mice

**Figure 5:**
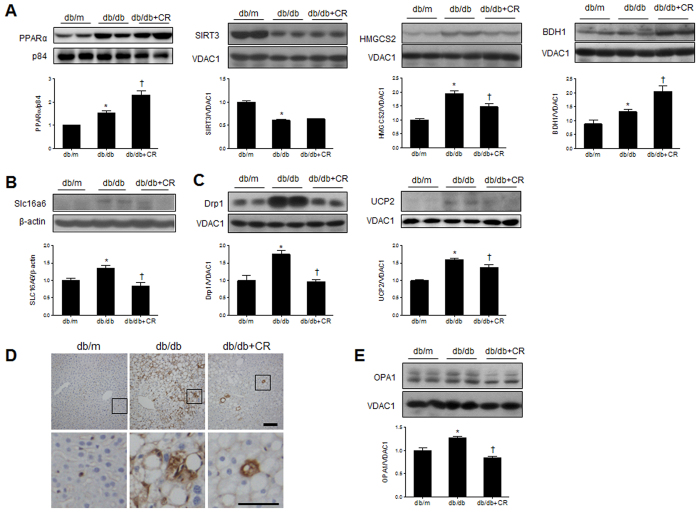
Effects of caloric restriction (CR) on ketogenesis and mitochondrial dysfunction in the livers of db/db mice. (**A**) Western blots and quantification showing ketogenesis-related mitochondrial enzymes PPARα, SIRT3, HMGCS2, and BDH1. (**B**) Western blots and quantification showing hepatic Slc16a6 expression. (**C**) Western blots and quantification showing hepatic mitochondrial Drp1 and UCP2 expression, with band intensity normalized to VDAC1. (**D**) Immunohistochemistry detecting Drp1 in liver sections. Scale bar, 100 μm. (**E**) Western blots and quantification showing hepatic mitochondrial OPA1 expression. Data are shown as the mean ± SEM. *p < 0.05 for db/db versus db/m mice. ^†^*p* < 0.05 for db/db+CR versus db/db mice.

To determine the effects of CR on mitochondrial β-oxidation and ketogenesis, we examined the expression of peroxisome proliferator-activated receptor α (PPARα), sirtuin 3 (SIRT3), 3-hydroxy-3-methylglutaryl CoA synthase 2 (HMGCS2), and 3-hydroxybutyrate dehydrogenase, type 1 (BDH1) in mitochondria from these mice (Fig. 5A). PPARα is the master regulator of fatty acid β-oxidation and ketogenesis^20^, while SIRT3 deacetylates and activates mitochondrial HMGCS2, promoting ketogenesis in the fasted state^21^. We showed that PPARα and SIRT3 expression were significantly increased and decreased, respectively, in db/db mice compared to db/m mice. However, CR did not change their expressions. In particular, only the HMGCS2 level was significantly increased in db/db mice compared to db/m mice, while its expression in mitochondria was reduced by CR. Unlike HMGCS2, the increased BDH1 level in db/db mice was not reduced by CR. Finally, we determined the effect of CR in db/db mice on hepatic Slc16a6 (Fig. 5B), which acts as a ketone body transporter in the liver during fasting^22^. We showed that induction of Slc16a6 in db/db mice was inhibited by CR.

Mitochondrial dysfunction contributes to the pathogenesis of NAFLD since it affects hepatic lipid homeostasis and promotes reactive oxygen species (ROS) production and lipid peroxidation, cytokine release, and cell death^23^. To evaluate the effect of CR on mitochondrial biogenesis, we examined the expression of mitochondrial fission- and fusion-related proteins [dynamin-related protein 1 (Drp1) and optic atrophy 1 (autosomal dominant) (OPA1)] and a mitochondrial carrier protein [uncoupling protein 2 (UCP2)] (Fig. 5C–E). Drp1 and UCP2 expression was higher in db/db mice than in db/m mice, while their levels were decreased by CR (Fig. 5C). Immunohistochemical staining showed that CR attenuates the increase of Drp1-positive hepatocytes in the liver of db/db mice (Fig. 5D). In addition, we found that mitochondrial fusion-related protein OPA1 was significantly decreased by CR (Fig. 5E).

### Effects of CR on inflammation, collagen deposition, and ER stress in the livers of db/db mice

**Figure 6:**
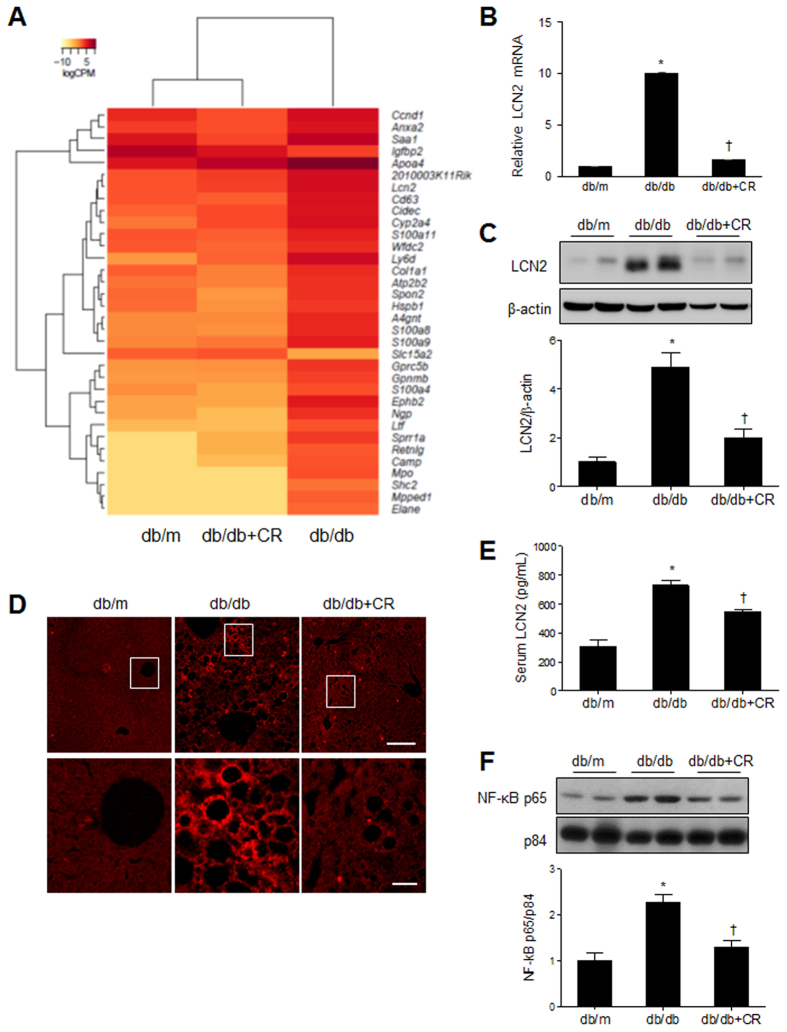
Effects of caloric restriction (CR) on inflammation in the livers of db/db mice. (**A**) The differential expression of genes in WT or db/db+CR versus db/db mice was color-shaded after NGS-based RNA-seq analysis. Genes shown in red have up-regulated expression and those shown in yellow have down-regulated expression (Table S3). (**B**) Quantitative RT-PCR analysis of LCN2 in the liver. (**C**) Western blots and quantifications showing hepatic LCN2 expression (band intensity normalized to β-actin). (**D**) Representative immunofluorescent images of LCN2 in liver sections (scale bar, 100 μm). (**E**) Serum LCN2 levels. (**F**) Western blots and quantifications showing nuclear NF-kBp65 expression (band intensity normalized to p84). Data are shown as the mean ± SEM. *p < 0.05 for db/db versus db/m mice. ^†^*p* < 0.05 for db/db+CR versus db/db mice.

As shown in Fig. 3, we found that CR reverses inflammation-related metabolites in db/db mice. To identify molecules responsible for obesity/diabetes-induced inflammation, we conducted next generation sequencing (NGS)-based RNA-seq analysis and examined hepatic gene expression profiles in db/m and db/db mice with or without CR (Fig. 6A). We identified 44 differentially expressed genes (DEGs) (*p* < 0.01), listed in Table S3. In particular, we found one gene, lipocalin-2 (LCN2), which regulates intracellular lipid droplet formation in the liver and is closely associated with inflammation during NAFLD^24^. We further examined LCN2 mRNA and protein expression in the liver, and found it was increased in db/db mice and reduced by CR (Fig. 6B,C). Immunofluorescence staining showed that CR attenuates the increase of LCN2-positive hepatic stellate cells in the liver of db/db mice (Fig. 6D). We found that CR attenuated increased serum LCN2 levels in db/db mice (Fig. 6E). In support of the anti-inflammatory effect of CR, we confirmed that CR inhibited the nuclear translocation of nuclear factor-kappa B (NF-κb) p65 (Fig. S7). Western blot analysis showed that an increase in n uclear NF-κb p65 expression in db/db mice is also decreased by CR (Fig. 6F).

Furthermore, to investigate whether CR affects hepatic collagen deposition in db/db mice with hepatic inflammation, we examined connective tissue growth factor (CTGF), collagen content, and alpha-smooth muscle actin (α-SMA) (Fig. S8A–C). Western blot analysis showed that hepatic CTGF expression was significantly higher in db/db mice than db/m mice, whereas its levels were significantly decreased by CR (Fig. S8A). Using the Sircol collagen assay, we found that CR counteracted the increase in hepatic collagen in db/db mice (Fig. S8B). Finally, immunohistochemistry showed that immunoreactivity of α-SMA-positive cells in the liver of db/db mice was decreased by CR (Fig. S8C). These findings indicate that CR may inhibit the development of hepatic steatosis into hepatic profibrotic state.

Hepatic TG can increase the accumulation of unfolded proteins in the ER, which leads to ER stress in many metabolic diseases, including obesity, T2D, and NAFLD^25^. We finally examined the effects of CR on the protein levels of some ER stress markers in the liver of db/db mice (Fig. S8D). These markers [protein kinase RNA-like endoplasmic reticulum kinase (PERK), phospho-elF2α (p-elF2α), activating transcription factor 4 (ATF4), and CCAAT-enhancer-binding protein homologous protein (CHOP)] were significantly increased in db/db mice compared to db/m mice. Long-term CR administration significantly inhibited the increase in these ER stress markers.

## Discussion

We demonstrate here that CR is a beneficial therapy to attenuate the deleterious actions of NAFLD including lipogenesis, ketogenesis, autophagy, mitochondrial biogenesis, inflammation, collagen deposition, and ER stress in db/db mice. In mice with an obese phenotype with fully developed T2D, we generated metabolite profiles of NAFLD and identified several metabolites that indicate lipogenesis, ketogenesis, and inflammation reversed by CR. Our findings support that the metabolic pathways leading to the development of hepatic steatosis are multiple and include enhanced non-esterified fatty acid release from adipose tissue (lipolysis), increased de novo fatty acids (lipogenesis), decreased β-oxidation and mitochondrial fission in the liver. Our findings were consistent with previous results from humans^10^,^11^,^26^ and we focused on the effects of CR in NAFLD for these metabolic changes.

NAFLD is characterized by lipogenesis, disruption of autophagy, and accumulating glycerolipids in the liver with obesity and diabetes^27^,^28^. Consistent with previous evidence that increased SIRT1 expression represses SREBP-1 and inhibits lipid synthesis and fat storage^29^, hepatic SIRT1 expression was significantly decreased in db/db mice compared to db/m mice. Likewise, our results showed that CR inhibits expression of the proteins related to lipogenesis (FAS, SCD1, LXR β, SREBP-1, and DGAT1) and energy metabolism (phosphorylation of AMPK and ACC) by increasing SIRT1. These data indicate that CR inhibits the accumulation of glycerolipids in the liver of db/db mice and that they are decomposed to FFAs by enhancing autophagy. Autophagy has been identified to regulate intracellular lipid stores through degradation of lipid droplets and release of FFA as a rapid response to starvation^30^. Chronic high-fat diet (HFD) feeding of mice, which induces insulin resistance and hepatic steatosis, impairs lipid autophagy and expression of several autophagy proteins in the liver^31^. Autophagy-related proteins are decreased in the liver of ob/ob mice^32^. Like those of ob/ob mice, we showed that hepatic LC3B expression levels were decreased in db/db mice, but the p62 level, which is related to the inhibition of autophagy was increased in db/db mice. These altered genes may cause an increase in ER stress, similar to that of autophagy deficiency in db/db mice. In contrast, levels of hepatic LC3B and p62 expressions are reversed by CR.

SREBP-1, ChREBP, and LXR in the liver are associated with transcriptional regulation of TG synthesis^19^. SREBP-1 and ChREBP are required for transcriptional control by insulin and glucose, respectively^33^. These two transcriptional factors efficiently respond to carbohydrates. However, we found that hepatic ChREBP expression in db/db mice was not reversed by long-term CR. The activation of ChREBP regulated by glucose contributes to translocation from the cytosol into the nucleus^34^. We found that nuclear ChREBP localization in the liver of db/db mice was increased compared to that in db/m mice. However, its expression was not inhibited by CR. Thus, we hypothesize that ChREBP may play an important role in hepatic lipogenesis in NAFLD. In support of this, db/db mice are characterized by severe hyperglycemia with loss of beta cells^35^. For the reason that CR did not reduce serum FFA levels in db/db mice, the clearance from increased lipolysis in adipose tissues and released FFA into circulation may be not completely done by CR for 12 weeks. These data indicate that the induction of lipogenic genes is under the control of SREBP-1 and ChREBP in response to insulin and glucose, respectively. However, glucose-dependent ChREBP is not controlled by CR.

PPARα is mainly present in the liver and regulates the expression of genes that encode for enzymes involved in fatty acid transport, lipid binding and activation, and peroxisomal and mitochondrial fatty acid β-oxidation^36^. PPARα activation also stimulates de novo hepatic lipogenesis^37^. PPARα increases β-oxidation and gluconeogenesis during fasting^4^. In accordance with our results, PPARα mRNA levels are increased in streptozotocin-induced rat liver, ob/ob, and db/db mice^38^,^39^. However, we found that increased nuclear PPARα levels in the livers of db/db were not inhibited by CR. As shown in Table 1, circulating FFA levels in db/db mice were not decreased by long-term CR. We suggest that increased serum FFA from lipolysis of adipose tissues in db/db+CR mice may play an important role in the transcriptional activity of PPARα in the liver. In addition, these data indicate that glycerolipids are decomposed to FFAs and immediately oxidized or exported to the systemic circulation as an energy source during CR.

SIRT3 is localized in mitochondria and plays an important role in mitochondrial metabolism^40^. SIRT3 is increased during fasting, including CR, and decreased in obese mice^41^. We also found that mitochondrial SIRT3 expression was significantly reduced in the liver of db/db mice compared to that of db/m mice. However, hepatic SIRT3 expression in db/db mice could be not reversed by CR. Although CR significantly decreases β-hydroxybutylate in the liver of db/db mice, it could not attenuate the increased serum FFA and cholesterol levels of db/db mice. These data indicate that entry of excessive circulating FFAs into mitochondria in the liver can contribute to the reduction of mitochondrial SIRT3 levels in both db/db and db/db+CR mice.

HMGCS2 is activated by mitochondrial deacetylase SIRT3^42^. FFA oxidation is the major fuel for ketone bodies (e.g., acetoacetate, acetone, and β-hydroxybutyrate) which are mainly used under conditions of prolonged fasting, illness, or increased physical activity^43^. Obesity induces unusual lipid oxidation and ketone body production^44^. Consistently, like the levels of metabolites (acetone and β-hydroxybutyrate), the level of mitochondrial HMGCS2 was increased in db/db mice, while it was significantly decreased by CR. However, the mitochondrial BDH1 level in db/db mice was augmented by CR. By contrast, interconversion of acetoacetate and β-hydroxybutyrate by BDH1 appears to be readily reversible. BDH1, which contains several SIRT3-regulated acetylation sites, is not affected by SIRT3, and its mitochondrial level in db/db mice cannot be changed by CR. Therefore, we expected that many ketone bodies from fatty livers were produced for energy sources. However, these mice could not use glucose as energy because of insulin resistance and increasing gluconeogenesis.

Slc16a6 is known as a selective β-hydroxybutyrate transporter required in the liver during fasting, that causes impairment of hepatic ketone body secretion^22^. We suggest that the accumulation of ketone bodies within the fatty liver of db/db mice leads to an increase in Slc16a6 levels within the liver as a mechanism for adaptation to this increased secretion of ketone bodies.

In addition to ketogenesis from β-oxidation in mitochondria, abnormality of mitochondrial dynamics is associated with enhanced fission in the liver of db/db mice. Consistently, we found that mitochondrial fission-related enzyme Drp1 was increased in the liver of db/db mice and reduced by CR. This suggests that mitochondrial fission induced by hepatic damage is reversed by CR. Expression of fusion-related protein OPA1 also increased in db/db mice, likely to maintain mitochondrial homeostasis. UCP2 was also up-regulated in the liver of db/db mice, which is a potential regulator of mitochondrial ROS production^28^,^45^. Our results indicate that CR partially attenuates obesity-induced mitochondrial fragmentation and ROS production.

In db/db mice, long-chain fatty acids (LCFAs) are oxidized in peroxisomes and the ER in place of mitochondria, because of carnitine palmitoyltransferase 1 (CPT1) inhibition^43^. This increased FFA oxidation in the ER causes ER stress^46^. ER stress is evident in the liver of obese mice and plays a critical role in the development of insulin resistance and diabetes^47^. We found that enzymes related to ER stress, such as PEPK, P-elF2a, ATF, and CHOP, were activated in db/db+CR mice. We hypothesized that ROS induced by NAFLD contribute to lipotoxicity in hepatocytes. Levels of glutathione and ascorbate antioxidants were increased in db/db mice. These results suggest that CR reduces the load for protein folding in the ER in db/db mice, and thus reduces the activity of PERK followed by regulation of the elF2α-ATF4 axis in the liver. As a result, ROS from ER stress is reduced and major lipids in the membrane (PC, PE, and SM) are restored. Improvement of the lipid membrane may protect mitochondria from lipotoxicity and increase fat metabolism, by reducing glycerolipids in hepatocytes.

**Figure 7:**
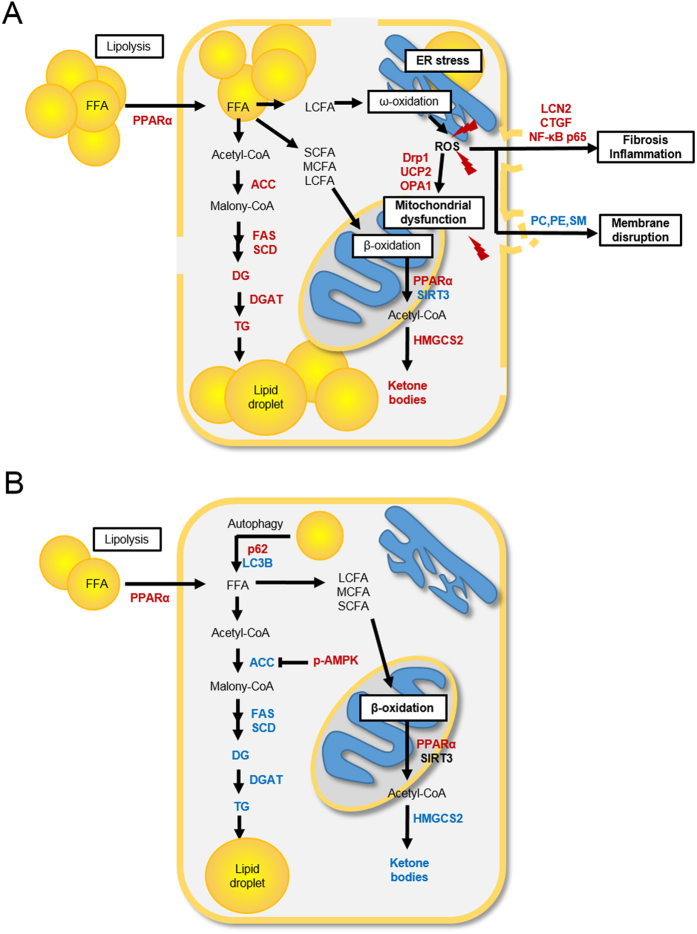
Changes in hepatocyte metabolism in non-alcoholic fatty liver disease (NAFLD) and caloric restriction (CR) states. Increased metabolites and enzymes are shown in red and decreased in blue. Non-detection or no change is shown in black. (**A**) NAFLD model compared with the db/m model. (**B**) CR model compared with the NAFLD model.

In addition to examining autophagy and ER stress in the liver of db/db mice, we confirmed the beneficial effect of CR on inflammation. Obesity and T2D are closely associated with chronic inflammation characterized by an abnormal cytokine production and the activation of a network of inflammatory signaling pathways^48^. We found that glycerolphospholipids and sphingolipids in db/db mice are significantly decreased by CR. Among these lipids, PC, PE, and SM are known to have a major role in hepatic cell membranes^27^,^46^,^49^. Kidd *et al*.^49^ have shown that ROS inactivates the membrane proteins that depend on the lipids for activity and weakens the membrane to the point of rupture. Other research has reported that inflammatory mediators activate SM-synthase and that SM-synthase makes SM and DG from ceramide and PC^50^. SM then accumulates in the outer membrane, and DG activates protein kinase C (PKC) and NF-κB, which are transcription factors of inflammation^50^. From our study, low levels of lipids (phospholipids and sphingolipids) and high levels of LCN2 and NF-κB (Fig. 7A–E) showed that the lipid membrane was decomposed and inflammatory response occurred in db/db mice. LCN2 is closely associated with obesity and T2D in humans^51^, and its gene expression is up-regulated in adipose tissue and the liver of genetically obese animals^52^. LCN2 induction is activated by the pro-inflammatory cytokine IL-1β, which is induced by the NF-κB pathway^53^. Consistently, we found that LCN2 and nuclear NF-κB p65 protein levels were increased in db/db mice compared to db/m mice, but reduced by CR. We also found that increased levels of CTGF and α-SMA expression in db/db mice were attenuated by long-term CR. Therefore, our findings indicate that obesity and diabetes-induced hepatic inflammation can be suppressed by CR, preventing the hepatic fibrosis that can occur as a result of chronic inflammation.

In conclusion, the present study explored the therapeutic effects of CR on NAFLD in db/db mice. We found that CR reduces obesity by suppressing the lipogenesis pathway in the liver of db/db mice. CR also improved hepatic steatosis, inflammation, and ER stress, and partially modulated mitochondrial dynamics. These results are summarized in Fig. 7, and suggest that CR may have beneficial effects on metabolic disorders. Our findings provide potential biomarkers for the multiple risk assessment of NAFLD and further insight into therapeutic strategy for NAFLD development.

## Methods

### Animals and the caloric restriction model

Five-week-old male db/db and db/m mice from the C57BL/6J background were purchased from Central Lab Animal, Inc. (Seoul, South Korea) and maintained in the animal facility at Gyeongsang National University. Experiments were performed in accordance with the National Institutes of Health Guidelines on the Use of Laboratory Animals (GNU-130306-M0021). All experimental protocols were approved by the ethics committee of Gyeongsang National University. Mice were individually housed with an alternating 12-h light/dark cycle. Starting at five weeks of age, mice were fed a normal standard diet chow (ND) for five weeks. Mice were then randomly divided into three groups, db/m, db/db and db/db+CR (n = 10 per group), at 10 weeks of age. For the db/db+CR group, db/db mice were transferred to individual cage and received a restricted amount of food (2 g/day) for 12 weeks as previously described^44^. The db/m and db/db mice were given free access to food. Mice were weighed four times monthly and immediately prior to their sacrifice at 22 weeks of age.

### Tissue collection and sample preparation

For histological evaluation, the mice (n = 3 per group) were anesthetized with zoletil (5 mg/kg, Virbac Laboratories, Carros, France) and transcardially perfused with heparinized saline and 4% paraformaldehyde. Six hours after fixing, the liver was processed for paraffin embedding and sectioned (5 μm). Liver sections were stained with hematoxylin and eosin (H&E) and visualized under a BX51 light microscope (Olympus, Tokyo, Japan). The histological analysis from three H&E stained liver sections each mice (n = 3 per group) was performed using the histological scoring system for NAFLD activity by an experienced pathologist without prior knowledge of the groups. The NAFLD activity score was quantified by summing the scores of steatosis (0–3), lobular inflammation (0–2), and hepatocellular ballooning (0–2).

### Hepatic TG colorimetric assay

Frozen livers were homogenized and centrifuged, and the supernatants were used to determine triglyceride (TG) levels. TG concentrations (n = 7 per group) were measured by a TG colorimetric assay kit (Cayman Chemical Company, Ann Arbor, MI, USA).

### Oil Red O staining

To determine hepatic lipid accumulation, frozen liver sections (5 μm) were stained with 0.5% Oil Red O (Sigma–Aldrich, St. Louis, MO, USA) for 10 min, washed, and counterstained with Mayer’s hematoxylin (Sigma–Aldrich) for 45 sec. The sections were visualized under a BX51 light microscope (Olympus).

### Measurement of serum metabolic parameters

For serum analysis, the mice (n = 10 per group) were intramuscularly anesthetized with zoletil. Serum glucose, aspartate aminotransferase (AST), alanine aminotransferase (ALT), free fatty acids (FFA), total cholesterol, and triglyceride (TG) levels were determined at the Green Cross Reference Laboratory (Young, South Korea). Serum adiponectin, leptin, insulin, and lipocalin-2 (LCN2) were measured using mouse adiponectin, leptin, insulin (Shibayagi Co., Gunma, Japan), and LCN2 (R&D Systems, MN, USA) enzyme-linked immunosorbent assay (ELISA) kits.

### Metabolic profiling of liver tissue based on ^1^H-NMR and UPLC-QTOF-MS

For metabolite profiling of lipids and aqueous metabolites in liver tissue (n = 7 per group), ultra-performance liquid chromatography (UPLC) (Waters, Maidstone, UK) coupled to the quadrupole time of flight mass spectrometry (QTOF-MS) (ESI/Triple TOF 5600; SCIEX, Concord, ON, Canada) and ^1^H-nuclear magnetic resonance (^1^H-NMR) (Agilent Technologies Inc. Santa Clara, CA, USA) were used, respectively. For further details, see Supplementary data.

### Tissue fractionation and western blot analysis

For protein extraction (n = 7 per group), frozen liver tissue was homogenized in lysis buffer (15 mM HEPES [pH 7.9], 0.25 M sucrose, 60 mM KCl, 10 mM NaCl, 1 mM ethylene glycol tetraacetic acid, 1 mM phenylmethylsulfonyl fluoride, and 2 mM NaF). Homogenized tissues were incubated on ice for 20 min and sonicated. Samples were then centrifuged for 20 min at 12,000 rpm at 4 °C and supernatants were transferred to clean vials. For cytosolic and nuclear fraction preparations, livers were chopped in ice-cold lysis buffer (10 mM HEPES-KOH [pH 7.9], 1.5 mM MgCl_2_, 10 mM KCl, protease inhibitors), homogenized, and centrifuged for 1 min at 12,000 rpm. The supernatant was collected as a cytosol fraction and a nuclear pellet was resuspended in high-salt extraction buffer (20 mM HEPES-KOH [pH 7.9], 1.5 mM MgCl_2_, 420 mM NaCl, 0.2 mM EDTA, 25% glycerol, protease inhibitors, 0.5 mM DTT) and incubated on ice for 20 min, then centrifuged for 10 min at 12,000 rpm. The supernatant was collected as a nuclear fraction and centrifuged sequentially as described by Andrews and Faller^54^. For the separation of cytosolic and mitochondrial fractions, we used a mitochondrial isolation kit (Bio-Rad, Hercules, CA, USA). Samples were probed with primary antibodies (Table S1) and protein bands were detected using enhanced chemiluminescence substrates (Pierce, Rockford, IL, USA). The Multi-Gauge image analysis program (version 3.0; Fujifilm, Tokyo, Japan) was used for densitometry analysis.

### Immunohistochemistry

Deparaffinized sections of liver were placed in 0.3% H_2_O_2_ for 10 minutes, washed, and incubated in blocking serum for 20 min. Sections were incubated in primary antibodies (Table S1) at 4 °C overnight and with a secondary biotinylated antibody for 1 h at room temperature. After washing, sections were incubated in an avidin-biotin-peroxidase complex solution (Vector Laboratories, Burlingame, CA, USA) and developed with 0.05% diaminobenzidine (Sigma-Aldrich) containing 0.05% H_2_O_2_. The sections were then dehydrated in graded alcohols, cleared in xylene, and mounted under a coverslip with Permount (Sigma-Aldrich). Sections were visualized under a BX51 light microscope (Olympus).

### Next generation sequencing (NGS)-based RNA-seq analysis

C&K genomics (Seoul, South Korea) performed preparation of an RNA-seq library, sequencing, and bioinformatics analysis. Briefly, sequencing was performed by Illumina HiSeq2000 and the quality-filtered reads were aligned to the Mus_musculus genome (GRCm38) from the Ensembl database. The R package DESeq^55^ was used to find DEGs (*p* < 0.01), which were then converted to official gene symbols and grouped by a common biological property according to Gene Ontology (GO) and Kyoto Encyclopedia of Genes and Genomes (KEGG) pathway analyses. The enriched GO terms were used to functionally cluster DEGs, which were then filtered (*p* < 0.05). The RNA sequencing data from this study have been deposited under the NCBI Project Accession Number: PRJNA305333.

### Quantitative real-time reverse-transcription PCR (qRT-PCR)

Total RNAs were isolated using TRIzol reagent (Invitrogen, Carlsbad, CA, USA) and reverse-transcribed using the RevertAid™ First-Strand cDNA Synthesis Kit (Fermentas, Inc., Hanover, MD, USA). Real-time RT-PCR was performed using the ABI Prism 7000 Sequence Detection System (Applied Biosystems, Foster City, CA, USA). PCR amplifications were performed using the SYBR Green I qPCR kit (TaKaRa, Shiga, Japan) with specific primers: 5′-CCCCATCTCTGCTCACTGTC-3′ and 5′-TTTTTCTGGACCGCATTG-3′ for mouse *LCN2* (GenBank: NM_008491), and 5′-TGACCACAGTCCATGCCATC-3′ and 5′-GACGGACACATTGGGGGTAG-3′ for mouse *Gapdh* (GenBank: NM_001289726).

### Statistical analysis

A multivariate statistical analysis was performed using SIMCA-P+ software (version 12.0, Umeå, Sweden). Partial least-squares discriminant analysis (PLS-DA) was conducted for model discrimination. Score plots, loading plots, and variable importance of projection (VIP) values were obtained from the PLS-DA model. For further details, see Supplementary data.

## Additional Information

**How to cite this article**: Kim, K. E. *et al*. Caloric restriction of db/db mice reverts hepatic steatosis and body weight with divergent hepatic metabolism. *Sci. Rep.* **6**, 30111; doi: 10.1038/srep30111 (2016).

## Supplementary Material

Supplementary Information

## Figures and Tables

**Table 1:** Serum metabolic parameters in db/db mice with or without CR.

Metabolic parameters	db/m (n = 10)	db/db (n = 10)	db/db+CR (n = 10)
Insulin (ng/mL)	0.98 ± 0.22	5.43 ± 1.94*	1.17 ± 0.24†
Leptin (ng/mL)	15.22 ± 1.79	41.09 ± 4.08*	26.60 ± 2.92†
Adiponectin (μg/mL)	8107.44 ± 848.93	7685.24 ± 669.70	9925.84 ± 414.03†
AST (U/L)	77.20 ± 8.15	248.89 ± 48.60*	120.80 ± 16.01†
ALT (U/Ll)	29.00 ± 1.62	234.00 ± 54.77*	66.40 ± 11.14†
Glucose (mg/dL)	396.50 ± 30.68	864.00 ± 87.52*	851.10 ± 69.01
Total cholesterol (mg/dL)	93.00 ± 7.48	214.67 ± 18.05*	98.40 ± 7.82†
Triglyceride (mg/dL)	70.60 ± 7.76	163.89 ± 11.71*	123.10 ± 13.34†
Free fatty acid (μEq/L)	1100.00 ± 101.85	1930.89 ± 206.23*	1839.50 ± 152.69

Data are presented as the mean ± SEM. ^*^P < 0.05 for db/db mice vs. db/m mice, ^†^*P* < 0.05 for db/db+CR vs. db/db mice. AST, aspartate aminotransferase; ALT, alanine aminotransferase; CR, caloric restriction.
