# Supplementary material for: Caloric restriction of db/db mice reverts hepatic steatosis and body weight with divergent hepatic metabolism
Source: Sci Rep. 2016 Jul 21;6:30111. doi: 10.1038/srep30111 (PMC4954985; doi:10.1038/srep30111)
Supplement: Supplementary Information [file srep30111-s1.pdf]

**Caloric restriction of db/db mice reverts hepatic steatosis and body weight with divergent hepatic metabolism**

Kyung Eun Kim [1,*], Youngae Jung[2,*], Soonki Min[2,3,*], Miso Nam[2,3], Rok Won Heo[1], Byeong Tak Jeon[4], Dae Hyun Song[5], Chin-ok Yi[1], Eun Ae Jeong[1], Hwajin Kim[1], Jeonghyun Kim[6], Seon-Yong Jeong[6], Woori Kwak[7], Do Hyun Ryu[3], Tamas L. Horvath[8], Gu Seob Roh[1,8,†] and Geum-Sook Hwang[2,9†]

[1]Department of Anatomy and Convergence Medical Science, Bio Anti-aging Medical Research Center, Institute of Health Sciences, Gyeongsang National University School of Medicine, Jinju, Republic of Korea

[2]Integrated Metabolomics Research Group, Western Seoul Center, Korea Basic Science Institute, Seoul, Republic of Korea

[3]Department of Chemistry, Sungkyunkwan University, Suwon, Republic of Korea

[4]Department of Biochemistry, University of Nebraska-Lincoln, Lincoln, NE 68588, USA

[5]Department of Pathology, Institute of Health Sciences, Gyeongsang National University School of Medicine, Jinju, Republic of Korea

[6]Department of Medical Genetics, Ajou University School of Medicine, Suwon, Republic of Korea

[7]C&K Genomics, Seoul, Republic of Korea

[8]Program in Integrative Cell Signaling and Neurobiology of Metabolism, Section of Comparative Medicine, Yale University School of Medicine, New Haven, CT 06520, USA

[9] Department of Chemistry and Nano Science, Ewha Womans University, Seoul, Republic of Korea

*These authors contributed equally to this work.

†Correspondence should be addressed to:

Gu Seob Roh, M.D., Ph.D.

Department of Anatomy and Convergence Medical Science, Bio Anti-aging Medical Research Center, Gyeongsang National University School of Medicine, 15, Jinju-daero 816 Beon-gil, Jinju-si, Gyeongnam, 52727, Republic of Korea. Tel: +82-55-772-8035; Fax: +82-55-772-8039, E-mail: anaroh@gnu.ac.kr

Geum-Sook Hwang, Ph.D.,

Integrated Metabolomics Research Group, Western Seoul Center, Korea Basic Science Institute, Seoul 120-140, Republic of Korea. Tel: +82-2-6908-6200; Fax: +82-2-6908-6239, E-mail: gshwang@kbsi.re.kr

## Materials and Methods

### *Blood glucose and the insulin tolerance test (ITT)*

Blood glucose was measured every two weeks using an Accu-Chek glucometer (Roche Diagnostics GmbH, Mannheim, Germany). For the ITT, mice were given intraperitoneal injections of insulin (0.75 U/kg, Humulin-R; Eli Lilly, Indianapolis, IN, USA), and blood samples were collected before and 15, 30, 45, and 60 minutes after the injection. Blood glucose was measured using an Accu-Chek glucometer (Roche Diagnostics).

### *Sircol collagen assay*

The Sircol collagen assay is a quantitative dye-binding method for the analysis of acid and pepsin-soluble collagens. The collagen concentration from frozen liver tissues was determined by using a Sircol assay kit (Bioclor Ltd., Northern Ireland, UK).

### *Immunofluorescence*

For immunofluorescence staining, deparaffinized sections from the liver were incubated with goat anti-LCN2 or mouse anti-NF-Kb at 4°C for one day. After washing three times with 0.1 M PBS, sections were incubated with Alexa Fluor 594-conjugated donkey anti-goat or mouse secondary antibody (Invitrogen, Carlsbad, CA, USA). Nuclei were stained with DAPI (1:10,000, Invitrogen). Fluorescence was visualized under a BX51-DSU microscope (Olympus).

### *Metabolite analysis of liver tissue*

For analyzing both aqueous and organic metabolites, we followed a slightly modified two-step solvent extraction [1]. Frozen liver tissues (80 mg) were loaded into appropriate bead beating tubes. Then, 1.5 ml

of pre-chilled methanol:water (1:1, v:v) was added. For extraction, samples were homogenized twice at 5,000 rpm for 10 min and centrifuged at 16,000 g for 10 min. After centrifuging, the supernatant of the aqueous phase was transferred to an Eppendorf tube and completely dried using a speed vacuum. Then, 1.6 ml of dichloromethane:methanol (1:1, v:v) were added to remaining solid precipitates. Samples were homogenized as before, followed by centrifugation. Then, the supernatant of the organic phase was transferred to a new tube and dried under a stream of oxygen-free $N_2$.

***$^{1}$H-NMR measurements of polar metabolites***

For the NMR experiment, each extract was dissolved in a 700-μl buffer solution (0.1 M phosphate buffer and pH 7.0), and the pH was adjusted to 7.0 ± 0.1. A 1-mM solution of 4,4-dimethyl-4-silapentane-1-sulfonic acid (DSS) dissolved in 99.8% $D_2O$ was added and a 600-μl aliquot was placed in a 5-mm NMR tube (Wilmad Lab Glass, Buena, NJ). The $^{1}$H-NMR spectra were acquired on a VNMRS-600 MHz NMR spectrometer (Agilent Technologies, Inc., Santa Clara, CA, USA) using a triple resonance 5-mm HCN salt-tolerant cold probe. The water-suppressed Carr-Purcell-Meiboom-Gill spin-echo pulse sequence [RD-90°-(τ-180°- τ) n-ACQ] with a total T2 relaxation time of 60 ms was used to attenuate broad signals from metabolites. For each sample, the $^{1}$H-NMR spectrum was collected with 128 transients into 65K data points using a spectral width of 12019.2 Hz with a relaxation delay of 2.0 s and an acquisition time of 2.7263 s. Free induction decays were weighted by an exponential function with a 0.3-Hz line-broadening factor prior to Fourier transformation. Signal assignment for representative samples was facilitated by acquisition of two-dimensional (2D) total correlation spectroscopy (TOCSY), correlation spectroscopy (COSY), H J-resolved (JRES), spiking experiments, and comparisons to literature.

***NMR data processing***

All spectra were phase-adjusted and baseline-corrected using the Chenomx NMR software suite (version 7.1, Chenomx, USA). The spectral region from $\delta$ = 0.12 to 9.35 was segmented into 0.001-ppm-wide

regions using the software. The regions corresponding to water (4.58–5.05 ppm), DSS (0.20–0.655 ppm, 1.645–1.808 ppm), lipid (1.246–1.360 ppm) and methanol (3.277–3.366 ppm) were excluded. Then, spectra were normalized to the total spectral area and converted to the ASCII format. ASCII files were imported into MATLAB (R2006a; Mathworks, Inc., Natick, MA, USA), and all spectra were aligned using the correlation optimized warping method. Identified metabolites were quantified using the Chenomx NMR software suite.

***UPLC-QTOF-MS measurements of lipid metabolites***

Dry residues of lipid extracts were dissolved in 200 μl of water:methanol (1:1, v:v) and filtered through the 0.2 μm hydrophilic PTFE syringe filter (MILLEX®, MILLIPORE). Then, 100-μl samples were diluted using 300 μl of isopropyl alcohol. An Acquity UPLC system (Waters, Maidstone, UK) including a binary solvent manager, column heater, and photodiode array detector was coupled with a hybrid Q-TOF tandem mass spectrometer (ESI/Triple TOF 5600; AB Sciex, Concord, ON, Canada). LC separation was carried out on BEH-C18 columns (100 mm × 2.1 mm, particle size 1.7 μm; Waters) on the reverse phase mode for 29 min. Column temperature and flow rate were set to 40°C and 0.35 ml/min, respectively. The mobile phase used was 10 mM ammonium acetate in acetonitrile:water (4:6, v:v) (A), and 10 mM ammonium acetate in acetonitrile:isopropyl alcohol (1:9, v:v) (B). The gradient elution program is as follows: 40–65% B at 0–5 min, 65–75% B at 5–20 min, 75–99% B at 20–25 min, 99% B for 2 min, 40% B at 27–27.1 min, and maintained for 1.9 min. Data acquisition was performed with a Triple TOF 5600 system with Turbo V sources and a Turbo ion spray interface. TOF-MS data were acquired both in positive and negative ion modes using an ion spray voltage of 4500 V. The nebulizer gas (Gas 1), heater gas (Gas 2), and curtain gas were set to 50 psi, 60 psi, and 30 psi, respectively. The Turbo spray temperature was 500°C and the declustering potential was ±90 V. Information-dependent acquisition (IDA) experiments were conducted to obtain single MS information and MS/MS information. TOF-MS and product ion calibrations were performed every day in both high-sensitivity and high-resolution modes

using an automated calibrant delivery system (CDS) before analysis. To check the reproducibility of the data, pooled quality control (QC) samples were measured per nine samples.

***UPLC-QTOF-MS data processing***

UPLC-QTOF-MS data from the liver extractions were processed by using MarkerView software version 1.2.1.1 (SCIEX, concord, ON, Canada) for peak finding and peak alignment. Data collection parameters in peak finding were set as follows: mass range, 100–1300 m/z; retention time range, 1.5–25.3 min; subtraction offset, 10 scans; subtraction multiplier, 1.8; minimum spectral peak width, 1 ppm; retention time peak width, 5 scans. For peak alignment, retention time and mass tolerance were set to 0.2 min and 10 ppm, respectively. The intensity threshold for peak filtering was set to 100, and peaks detected in fewer than four samples were removed. Peak area matrices were normalized by batch normalization to remove the systematic variation among the samples. The dataset resulted in peak lists containing the accurate mass of the precursor ion, retention time, and peak area of metabolites. To decrease instrumental bias including noise and variation, we eliminated peaks having a large variation in QC (coefficient of variation (CV)>20%). All of the variables were tentatively identified by connecting free accessible metabolite database such as HMDB (http://www.HMDB.ca) and Lipid Maps (http://www.lipidmaps.org) and comparing the literature.

***Statistical analysis***

A multivariate statistical analysis was performed using SIMCA-P+ software (version 12.0, Umeå, Sweden). Partial least-squares discriminant analysis (PLS-DA) was conducted for model discrimination. Score plots, loading plots, and variable importance of projection (VIP) values were obtained from the PLS-DA model. VIP>1 was considered to designate the most important metabolites responsible for the differentiation of groups. Student’s t-tests were performed using GraphPad Prism version 5.0 (GraphPad

Software, Inc., La Jolla, CA) to test the significance of differences in metabolite levels among groups. The differences were tested to a 95% probability level ($p$<0.05). For multiple comparisons, Bonferroni's corrections were applied ($p$<0.017). Differences in protein levels between groups were determined by one-way ANOVA, followed by *post hoc* analysis by using the Bonferroni's Multiple Comparison test. Results are presented as mean ± standard error of the mean (SEM), and $p$<0.05 was considered significant.

[1] Want EJ, Masson P, Michopoulos F, Wilson ID, Theodoridis G, Plumb RS, et al. Global metabolic profiling of animal and human tissues via UPLC-MS. Nature protocol 2013;8:17-32.

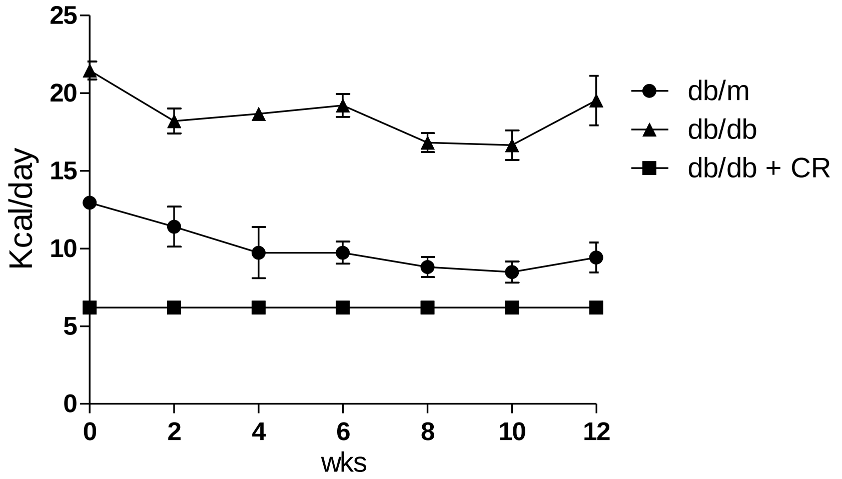


**Fig. S1. Effect of CR on total calorie intake (kcal) in db/db mice.** Total calorie intake (kcal) was calculated from the amount of food intake (g) by each group of mice.

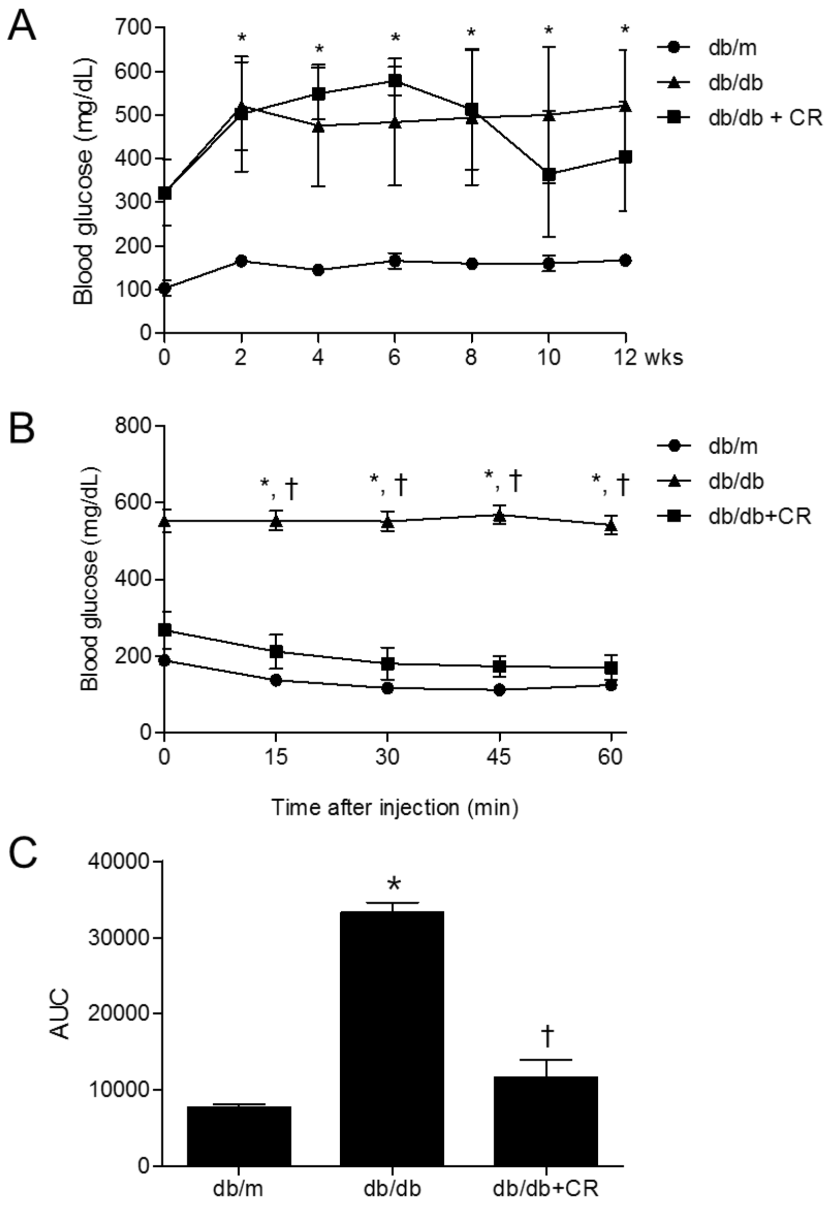


**Fig. S2. Effects of CR on blood glucose and insulin resistance in db/db mice.** (A) Fasting blood glucose levels. (B) Insulin tolerance test (ITT). Blood glucose levels after insulin treatment (0.75 U/kg). (C) Area under the curve (AUC) for ITT. Data are shown as the mean ± SEM. **p*<0.05 for db/db versus db/m mice. †*p*<0.05 for db/db+CR versus db/db mice.

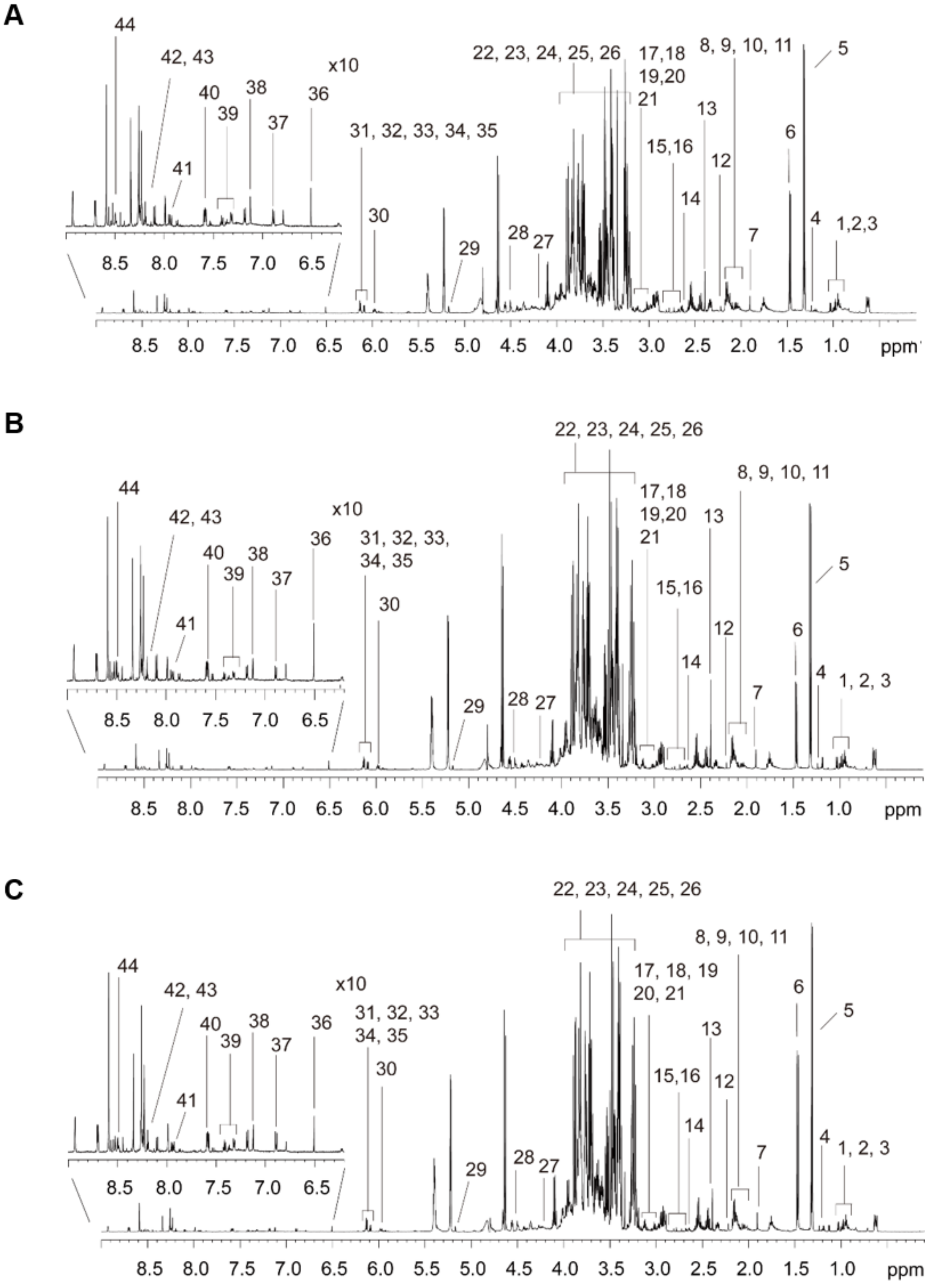


**Fig. S3**. **Representative $^{1}$H NMR Spectra of liver tissues extracted from three different groups.**

Db/m (A), db/db (B), and db/db+CR (C). Peaks: 1. Leucine; 2. Isoleucine; 3. Valine; 4. β-Hydroxybutyrate; 5. Lactate; 6. Alanine; 7. Acetate; 8. N-acetylglutamate; 9. Glutamate; 10. Glutathione; 11. Glutamine; 12. Acetone; 13. Succinate; 14. Methionine; 15. Dimethylamine; 16. Sarcosine; 17. Creatine; 18. Lysine; 19. Malonate; 20. Dimethylsulfone; 21. Choline; 22. Glucose; 23. Taurine; 24. Betaine; 25. Glycine; 26. Maltose; 27. Threonine; 28. Ascorbate; 29. Mannose; 30. UDP-Glucose 31. Adenosine; 32. Inosine; 33. AMP; 34. ATP; 35. ADP; 36. Fumarate; 37. Tyrosine; 38. Histidine; 39. Phenylalanine; 40. Niacinamide; 41. Xanthine; 42. Adenine; 43. Oxpurinol; 44. Formate.

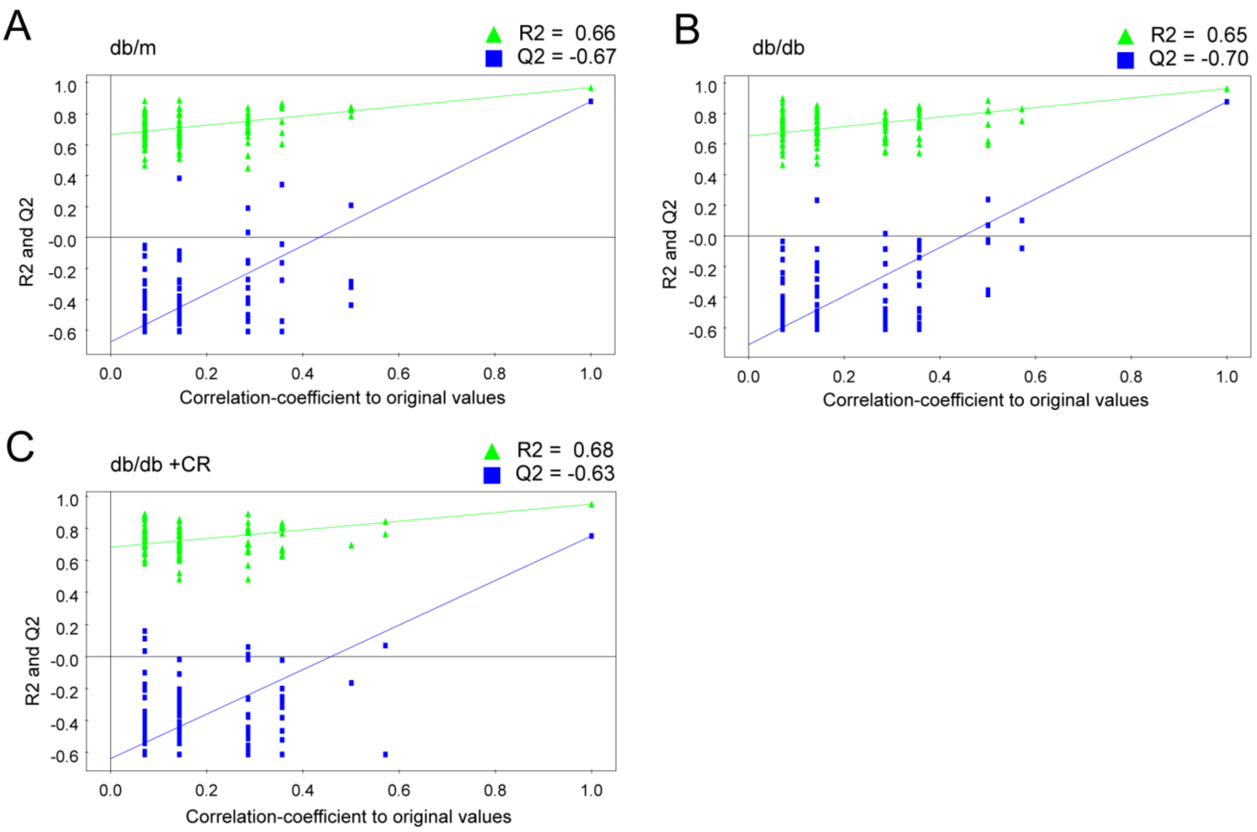


**Fig. S4. Validation plots of PLS-DA models derived from NMR spectra using a permutation test.** Permutation was randomly performed 100 times with the first principal component. The vertical axis represents R2 (goodness of fit) and Q2 (goodness of prediction) for every model, and horizontal axis represents the correlation coefficient between original and permuted response data.

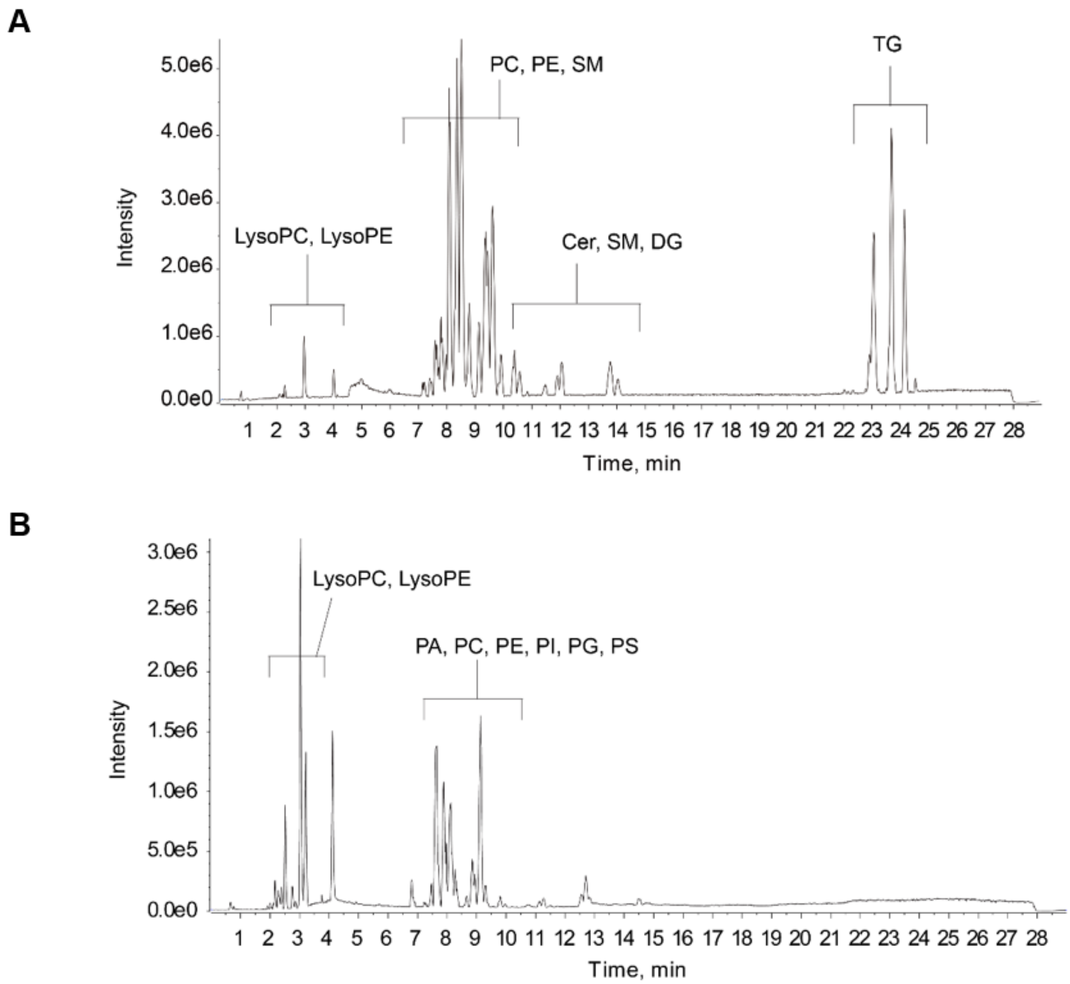


**Fig. S5. Representative base peak chromatogram of UPLC-QTOF-MS-positive (A) and -negative (B) ionization modes from mouse liver extracts.** Identified lipids are phosphocholine (PC), phosphatidylethanolamine (PE), lysoPC, lysoPE, sphingomyelins (SM), ceramide (Cer) diacylglycerol (DG), triacylglycerol (TG), phosphatidic acid (PA), phosphatidylinositol (PI), phosphatidylserine (PS), and phosphatidylglycerol (PG).

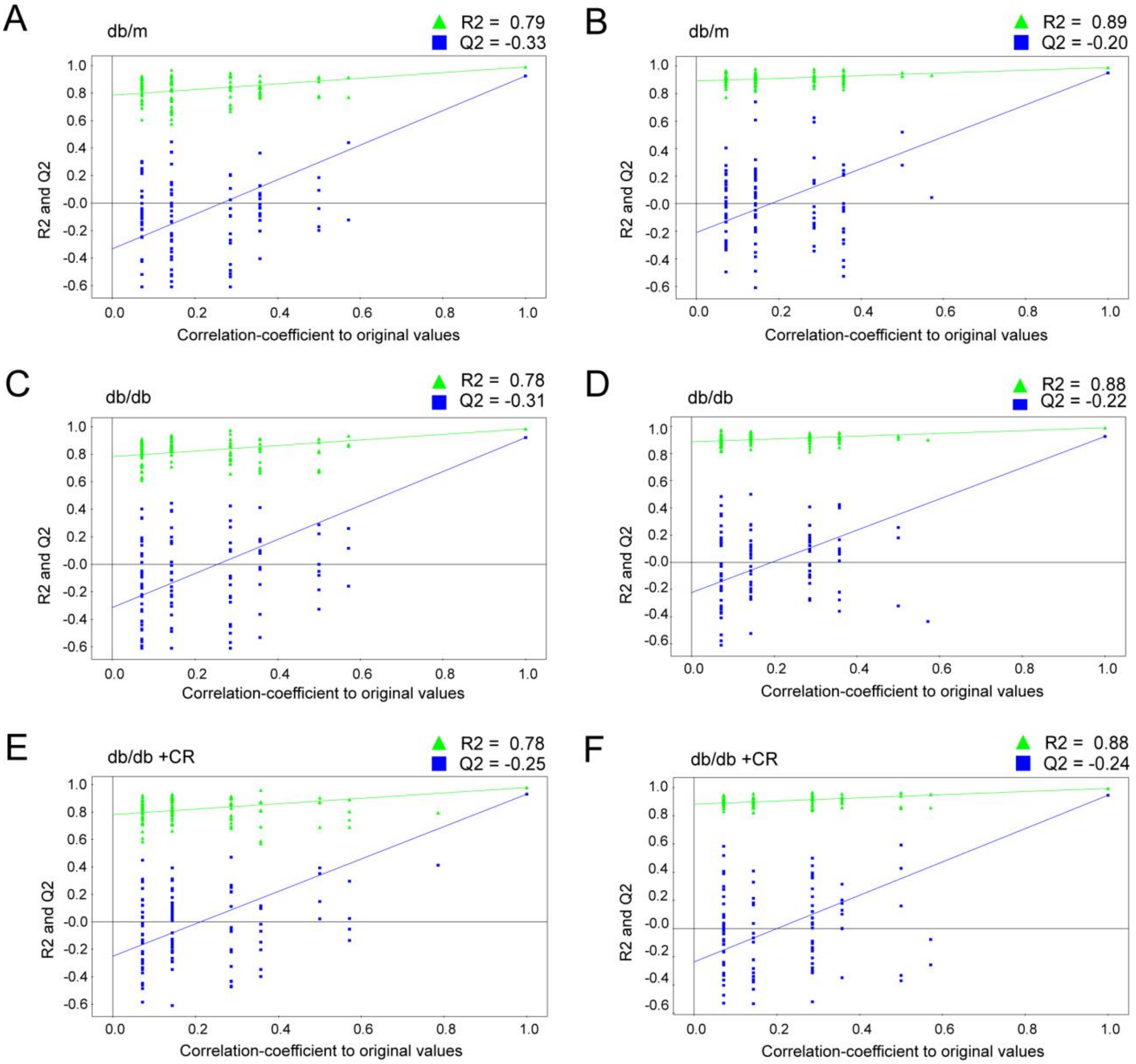


**Fig. S6. Validation plots of PLS-DA models derived from UPLC-QTOF-MS-positive (A, B, and C) and -negative (D, E, and F) ionization modes using a permutation test**. Permutation tests were randomly performed 100 times with the first principal component. The vertical axis represents R2 (goodness of fit) and Q2 (goodness of prediction) for every model, and horizontal axis represents the correlation coefficient between original and permuted response data.

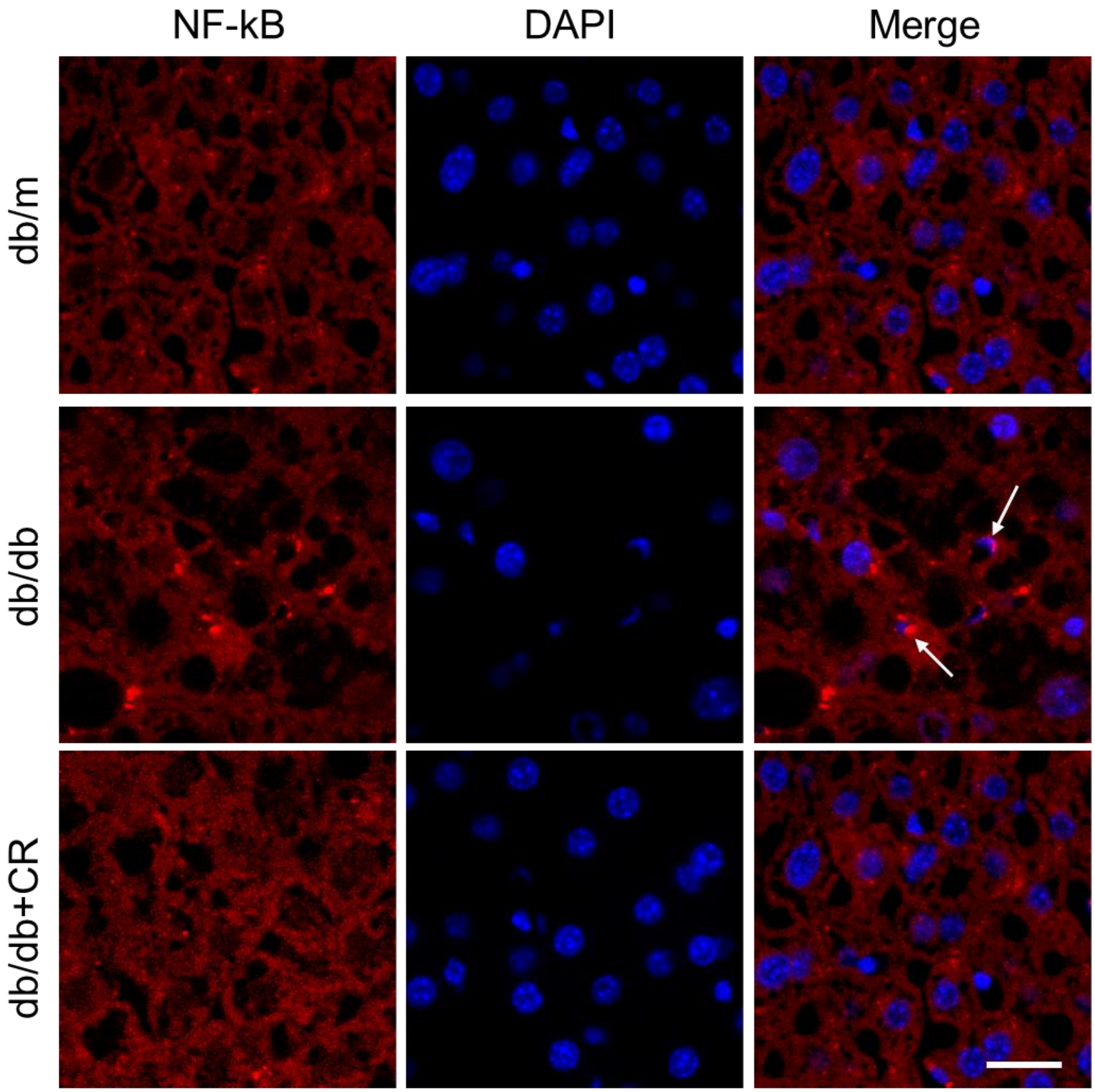


**Fig. S7. Effects of CR on nuclear translocaton in the livers of db/db mice.** Representative staining of NF-kB and DAPI in the liver sections. Arrows indicate hepatic stellate cell. Scale bar = 10 μm.

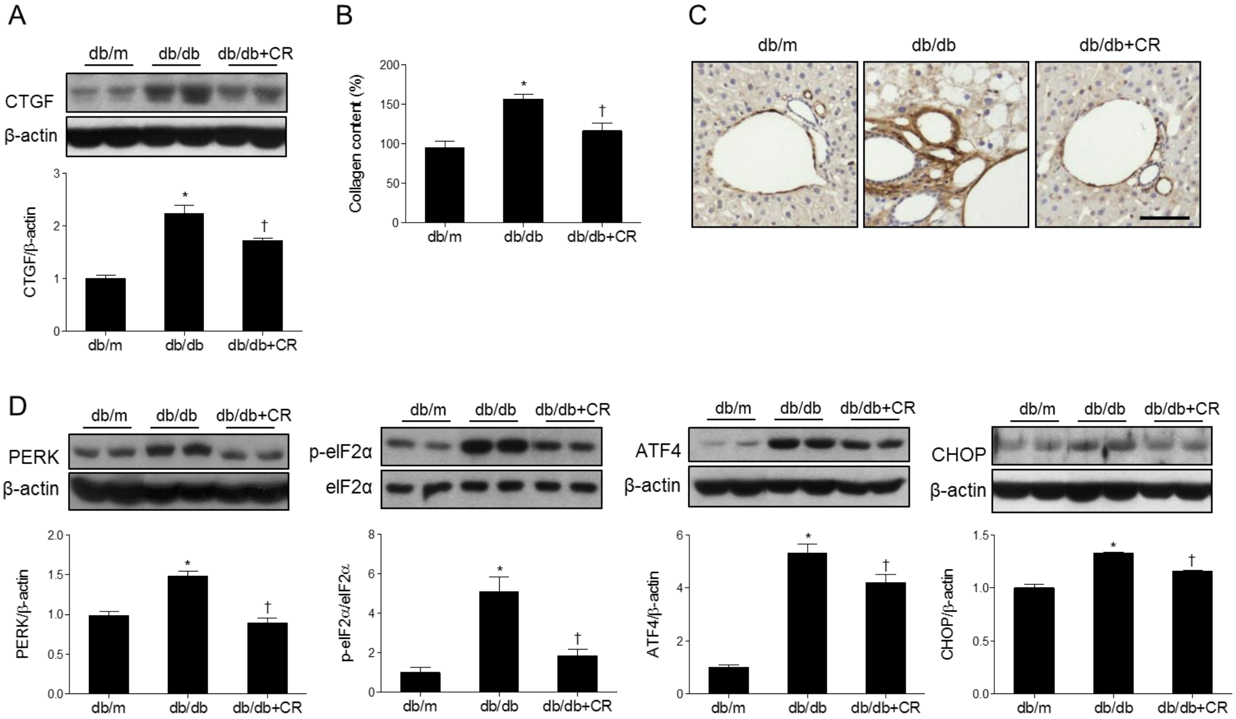


**Fig. S8. Effects of CR on collagen deposition and ER stress in the livers of db/db mice.** (A) Western blots and quantifications showing CTGF expression (with band intensity normalized to p84). (B) Hepatic collagen levels. (C) Immunohistochemistry for α-SMA detection in liver sections. Scale bar = 100 μm. (D) Western blots and quantifications showing hepatic PERK, p-elF2α, ATF4, and CHOP expression. Band intensity was normalized to β-actin or elF2α. Data are shown as the mean ± SEM. *$p$<0.05 for db/db versus db/m mice. †$p$<0.05 for db/db+CR versus db/db mice.

**Table S1. List of primary antibodies.**

| Antibody | Company | Catalog No. | Dilution(s) | Application(s) | Source |
|---|---|---|---|---|---|
| ACC | Cell signaling | #3662 | 1:1000 | WB | Rabbit |
| AMPK | Cell signaling | #2532 | 1:1000 | WB | Rabbit |
| ATF4 | Santa Cruz | sc-200 | 1:1000 | WB | Rabbit |
| α-SMA | Sigma | A5228 | 1:500 | IHC | Mouse |
| BDH1 | Santa Cruz | sc-99280 | 1:1000 | WB | Rabbit |
| CHOP | Santa Cruz | sc-575 | 1:1000 | WB | Rabbit |
| ChREBP | Novus | NB400-135 | 1:1000 | WB | Rabbit |
| CTGF | abcam | ab6992 | 1:1000 | WB | Rabbit |
| DGAT1 | Santa Cruz | sc-32861 | 1:1000 | WB | Rabbit |
| Drp1 | BD | 611113 | 1:1000, 1:100 | WB, IHC | Mouse |
| elF-2α | Cell signaling | #9722 | 1:1000 | WB | Rabbit |
| FAS | Cell signaling | #3189 | 1:1000 | WB | Rabbit |
| HMGCS2 | Genway | GWB-MV874A | 1:1000 | WB | Rabbit |
| LCN2 | R&D Systems | AF3508 | 1:1000, 1:200 | WB, IHC | Goat |
| LC3B | Sigma | L7543 | 1:1000 | WB | Rabbit |
| LXR β | R&D Systems | PP-K8917-00 | 1:1000 | WB | Mouse |
| NF-κB p65 | Cell signaling | #6956 | 1:1000 | WB, IHC | Mouse |
| OPA1 | BD | 612606 | 1:1000 | WB | Mouse |
| p-ACC | Cell signaling | #3661 | 1:1000 | WB | Rabbit |
| p-AMPK | Cell signaling | #2535 | 1:1000 | WB | Rabbit |
| p-elF-2α | Cell signaling | #3597 | 1:1000 | WB | Rabbit |
| PERK | Cell signaling | #3192 | 1:1000 | WB | Rabbit |
| PPAR α | abcam | ab8934 | 1:1000 | WB | Rabbit |
| P62 | Sigma | P0067 | 1:1000 | WB | Rabbit |
| SCD1 | Cell signaling | #2438 | 1:1000 | WB | Rabbit |
| SIRT1 | Cell signaling | #2028 | 1:1000 | WB | Rabbit |
| SIRT3 | abcam | ab189860 | 1:1000 | WB | Rabbit |

| | | | | | |
|---|---|---|---|---|---|
| Slc16a6 | Novusbio | NBP1-59881 | 1:10000 | WB | Rabbit |
| SREBP-1 | BD | 557036 | 1:1000 | WB | Mouse |
| UCP2 | Santa Cruz | sc-6525 | 1:1000 | WB | Goat |
| VDAC1 | abcam | ab15895 | 1:1000 | WB | Rabbit |
| p84 | abcam | Ab487 | 1:1000 | WB | Mouse |
| β-actin | Sigma | A5441 | 1:3000 | WB | Mouse |

WB, western blot; IHC, immunohistochemistry

**Table S2. Identification and quantification of metabolites from $^{1}$H NMR spectra obtained from liver extracts.**

| Metabolite | Chemical shift (ppm) | db/m | db/db | db/db+CR |
|---|---|---|---|---|
| 3-Hydrobutyrate [s] | 1.2(d), 2.3(d), 2.3(d), 4.1(m) | 0.058 ± 0.006 | 0.141 ± 0.016 * | 0.094 ± 0.009 † |
| Acetate [s] | 1.9(s) | 0.190 ± 0.015 | 0.279 ± 0.025 * | 0.196 ± 0.021 |
| Acetone [s] | 2.2(s) | 0.032 ± 0.003 | 0.049 ± 0.005 * | 0.039 ± 0.003 |
| Adenine [t, j] | 8.2(s), 8.2(s) | 0.039 ± 0.007 | 0.049 ± 0.009 | 0.058 ± 0.005 |
| Alanine [t] | 1.5(d), 3.8(q) | 2.009 ± 0.142 | 2.31 ± 0.303 | 2.813 ± 0.315 |
| AMP [s] | 4.0(m), 4.4(m), 4.5(q), 4.8(t), 6.1(d), 8.3(s), 8.6(s) | 0.728 ± 0.067 | 0.629 ± 0.088 | 0.616 ± 0.047 |
| Ascorbate [t] | 3.7(m), 4.0(m), 4.5(d) | 0.419 ± 0.021 | 0.664 ± 0.051 * | 0.36 ± 0.019 † |
| Betaine [s] | 3.3(s), 3.9(s) | 0.222 ± 0.019 | 0.186 ± 0.014 | 0.224 ± 0.03 |
| Choline [t, j] | 3.2(s), 3.5(m), 4.0(m) | 0.116 ± 0.009 | 0.114 ± 0.016 | 0.085 ± 0.007 |
| Creatine [s] | 3.0(s), 3.9(s) | 0.092 ± 0.005 | 0.072 ± 0.009 | 0.118 ± 0.022 |
| Dimethyl sulfone [s] | 3.1(s) | 0.040 ± 0.006 | 0.035 ± 0.003 | 0.055 ± 0.017 |
| Dimethylamine [s] | 2.7(s) | 0.008 ± 0.000 | 0.015 ± 0.001 * | 0.009 ± 0.001 † |
| Formate [s] | 8.5(s) | 0.086 ± 0.010 | 0.106 ± 0.013 | 0.105 ± 0.011 |
| Fumarate [s] | 6.5(s) | 0.095 ± 0.009 | 0.089 ± 0.015 | 0.095 ± 0.012 |
| Glucose [t] | 3.2(q), 3.4(m), 3.5(q), 3.7(m), 4.7(d), 5.2(d) | 20.31 ± 1.166 | 30.76 ± 1.205 * | 32.42 ± 1.115 |
| Glutamate [t, j] | 2.0(m), 2.1(m), 2.3(m), 2.4(m),3.8(q) | 0.879 ± 0.047 | 0.553 ± 0.049 | 0.733 ± 0.094 |
| Glutamine [t] | 2.1(m), 2.2(m), 2.4(m), 2.5(m), 3.8 (t), 6.9 (s) | 1.329 ± 0.059 | 1.686 ± 0.118 * | 1.650 ± 0.101 |
| Glutathione [s] | 2.1(q), 2.2(q), 2.5(m), 2.6(m), 2.9(q), 3.0(q), 3.8(m), 4.6(t), 8.2(s), 8.5(s) | 1.016 ± 0.036 | 1.429 ± 0.098 * | 1.113 ± 0.079 |
| Glycine [s] | 3.5(s) | 1.056 ± 0.064 | 0.737 ± 0.040 * | 0.822 ± 0.080 |
| Histidine [s] | 3.2(m), 4.0(q), 7.1(s), 7.8(s) | 0.206 ± 0.011 | 0.192 ± 0.012 | 0.194 ± 0.011 |
| Isoleucine [t] | 0.9(t), 1.0(d), 1.3(m), 1.5(m), 2.0(m), 3.7(d) | 0.099 ± 0.006 | 0.101 ± 0.004 | 0.086 ± 0.004 |
| Lactate [t, j] | 1.3(d), 4.1(q) | 4.171 ± 0.423 | 7.877 ± 0.937 * | 6.269 ± 0.731 |
| Leucine [t] | 0.9(d), 1.0(d), 1.7(m), 1.7(m), 1.7(m), 3.7(q) | 0.212 ± 0.022 | 0.245 ± 0.015 | 0.182 ± 0.012 † |
| Lysine [t] | 1.4(m), 1.5(m), 1.7(m), 1.9(m), 3.0(t), 3.7(t) | 0.109 ± 0.008 | 0.064 ± 0.013 | 0.064 ± 0.007 |
| Malonate [s] | 3.1(s) | 0.122 ± 0.020 | 0.113 ± 0.010 | 0.178 ± 0.053 |
| Maltose [t] | 3.3(q), 3.4(t), 3.6(q), 3.7(m), 3.8(m), 3.9(m), 4.0(t), 4.7(d), 5.2(d), 5.4(q) | 3.237 ± 0.391 | 2.382 ± 0.446 | 3.247 ± 0.625 |
| Mannose [t] | 3.4(m), 3.6(t), 3.7(m), 3.8(m), 3.9(m), 4.9(d), 5.2(d) | 0.479 ± 0.051 | 0.391 ± 0.046 | 0.486 ± 0.038 |
| Methionine [t, j] | 2.1(m), 2.2(m), 2.6(t), 3.9(q) | 0.086 ± 0.005 | 0.054 ± 0.005 * | 0.049 ± 0.005 |
| N-Acetylglutamate [s] | 1.9(m), 2.0(m), 2,2(t), 4.1(m), 8.0(d) | 0.032 ± 0.001 | 0.034 ± 0.005 | 0.024 ± 0.001 |
| Niacinamide [t, j] | 7.4(t), 7.6(m), 8.2(m), 8.7(q), 8.9(q) | 0.499 ± 0.033 | 0.476 ± 0.021 | 0.559 ± 0.030 |
| Oxpurinol [s] | 8.2(s) | 1.749 ± 0.064 | 1.411 ± 0.146 | 1.259 ± 0.099 |
| Phenylalanine [t] | 3.1(q), 3.3(q), 4.0(q), 7.3(d), 7.4(m) | 0.078 ± 0.006 | 0.073 ± 0.003 | 0.083 ± 0.007 |
| Sarcosine [s] | 2.7(s), 3.6(s) | 0.040 ± 0.005 | 0.039 ± 0.005 | 0.027 ± 0.002 |

| | | | | |
|---|---|---|---|---|
| Succinate [s] | 2.4(s) | 0.286 ± 0.039 | 0.413 ± 0.049 | 0.377 ± 0.037 |
| Taurine [t, j] | 3.3(t), 3.4(t) | 6.015 ± 0.491 | 7.400 ± 0.951 | 7.286 ± 0.846 |
| Threonine [t] | 1.3(d), 3.6(d), 4.2(q) | 0.356 ± 0.017 | 0.296 ± 0.009 * | 0.345 ± 0.015 |
| Tyrosine [s] | 3.0(q), 3.2(q), 3.9(q), 6.9(d), 7.2(d) | 0.132 ± 0.006 | 0.120 ± 0.008 | 0.130 ± 0.015 |
| UDP-Glucose [s] | 3.5(t), 3.5(m), 3.8(m), 3.9(m), 4.2(m), 4.3(m), 4.4(m), 5.6(q), 6.0(t), 7.9(d) | 0.090 ± 0.006 | 0.099 ± 0.011 | 0.105 ± 0.009 |
| Valine [t, b] | 1.0(d), 1.1(d), 2.2(m), 3.6(d) | 0.209 ± 0.012 | 0.214 ± 0.014 | 0.178 ± 0.008 |
| Xanthine [s] | 7.9(s) | 0.085 ± 0.006 | 0.089 ± 0.005 | 0.099 ± 0.095 |

Metabolites were identified by JRES (j), TOCSY (t) and spiking (s). Intensities are represented by the means ± SEM from n=7 samples per group. $p$<0.017 *db/m with db/db group, and †db/db+CR group by Mann-Whitney test.

**Table S3. List of down- and up-regulated genes in the liver of db/db mice with or without CR.**

| Gene Name | Gene Description | Expression in db/db | P-value (CR) | P-value (WT) |
|---|---|---|---|---|
| S100a4 | S100 calcium binding protein A4 | ↓ | 0.053 | 0.175 |
| Col1a1 | collagen, type I, alpha 1 | ↓ | 0.001 | 0.009 |
| Hspb1 | heat shock protein 1 | ↓ | 0.010 | 0.109 |
| Gprc5b | G protein-coupled receptor, family C, group 5, member B | ↓ | 0.002 | 0.000 |
| Mpo | myeloperoxidase | ↓ | 0.002 | 0.000 |
| Wfdc2 | WAP four-disulfide core domain 2 | ↓ | 0.055 | 0.055 |
| Elane | elastase, neutrophil expressed | ↓ | 0.058 | 0.013 |
| Shc2 | SHC (Src homology 2 domain containing) transforming protein 2 | ↓ | 0.188 | 0.065 |
| Retnlg | resistin like gamma | ↓ | 0.085 | 0.001 |
| Cd63 | CD63 antigen | ↓ | 0.000 | 0.000 |
| Lcn2 | lipocalin 2 | ↓ | 0.001 | 0.000 |
| S100a11 | S100 calcium binding protein A11 (calgizzarin) | ↓ | 0.010 | 0.002 |
| Ephb2 | Eph receptor B2 | ↓ | 0.000 | 0.000 |
| Gpnmb | glycoprotein (transmembrane) nmb | ↓ | 0.037 | 0.001 |
| Cidec | cell death-inducing DFFA-like effector c | ↓ | 0.002 | 0.000 |
| Atp2b2 | ATPase, Ca++ transporting, plasma membrane 2 | ↓ | 0.003 | 0.001 |
| Apoa4 | apolipoprotein A-IV | ↓ | 0.084 | 0.000 |
| Anxa2 | annexin A2 | ↓ | 0.006 | 0.007 |
| Ngp | neutrophilic granule protein | ↓ | 0.000 | 0.000 |
| Ltf | lactotransferrin | ↓ | 0.017 | 0.002 |
| Ly6d | lymphocyte antigen 6 complex, locus D | ↓ | 0.000 | 0.000 |
| Spon2 | spondin 2, extracellular matrix protein | ↓ | 0.000 | 0.005 |
| A4gnt | alpha-1,4-N-acetylglucosaminyltransferase | ↓ | 0.000 | 0.000 |
| Camp | cathelicidin antimicrobial peptide | ↓ | 0.016 | 0.000 |
| Mpped1 | metallophosphoesterase domain containing 1 | ↓ | 0.016 | 0.002 |
| 2010003K11Rik | RIKEN cDNA 2010003K11 gene | ↓ | 0.008 | 0.000 |
| Sprr1a | small proline-rich protein 1A | ↓ | 0.000 | 0.000 |
| S100a8 | S100 calcium binding protein A8 (calgranulin A) | ↓ | 0.000 | 0.000 |
| S100a9 | S100 calcium binding protein A9 (calgranulin B) | ↓ | 0.000 | 0.000 |
| Ccnd1 | cyclin D1 | ↓ | 0.000 | 0.035 |
| Saa1 | serum amyloid A 1 | ↓ | 0.000 | 0.045 |
| Cyp2a4 | cytochrome P450, family 2, subfamily a, polypeptide 4 | ↓ | 0.000 | 0.000 |
| Slc15a2 | solute carrier family 15 (H+/peptide transporter), member 2 | ↑ | 0.1055 | 0.0600 |
| Igfbp2 | insulin-like growth factor binding protein 2 | ↑ | 0.0172 | 0.0000 |

Up-regulated (↑) or down-regulated (↓) expression in db/db mice. *P* values for db/m or db/db+CR vs. db/db mice.
